# Impact of Octahedral Aluminum Sites on Guest Molecule Adsorption in Zeolites: A Computational Study of 5‐Fluorouracil in Zeolite FAU

**DOI:** 10.1002/chem.202500833

**Published:** 2025-05-02

**Authors:** Michael Fischer

**Affiliations:** ^1^ Faculty of Geosciences University of Bremen Klagenfurter Straße 2‐4 28359 Bremen Germany; ^2^ Bremen Center for Computational Materials Science (BCCMS) and MAPEX Center for Materials and Processes University of Bremen 28359 Bremen Germany

**Keywords:** density functional calculations, drug delivery, host‐guest interactions, molecular dynamics, zeolites

## Abstract

Many aluminosilicate zeolites contain octahedral aluminum sites, which may occur as extra‐framework or framework‐associated sites. Due to the Lewis acidity of these sites, their impact on catalytic properties has been investigated frequently. Comparatively less emphasis has been placed on their role in adsorption, despite evidence for an irreversible binding of some guest molecules like the anticancer drug 5‐fluorouracil (5‐FU) to octahedral Al atoms. In the present study, dispersion‐corrected density functional theory (DFT) calculations and DFT‐based ab initio molecular dynamics simulations (AIMD) are employed to investigate the adsorption of 5‐FU at a framework‐associated octahedral Al site in zeolite FAU. The calculations show that 5‐FU remains coordinated to the Al atom at room temperature and in the presence of water. In contrast, 5‐FU molecules adsorbed at framework protons are quickly displaced by water molecules. It is thus demonstrated that octahedral Al atoms will negatively affect the release of 5‐FU from zeolite hosts in drug delivery applications. A comparison of DFT‐calculated infrared (IR) spectra to literature data provides evidence that Al‐coordinated 5‐FU molecules were indeed present in previously investigated samples.

## Graphical abstract

Dispersion‐corrected density functional theory (DFT) calculations shed light on the interaction of the anticancer drug 5‐fluorouracil (5‐FU) with a framework‐associated octahedral aluminum site in a FAU‐type zeolite. The calculations help to explain the experimental observation of a limited 5‐FU release from zeolite samples containing such sites.

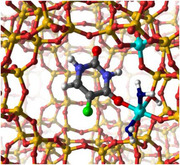

## 1 Introduction

The encapsulation of pharmaceutically active species in porous host materials can have a variety of benefits, such as increased bioavailability, enhanced solubility of poorly soluble drugs, and prolonged drug release, which can reduce side effects and enhance therapeutic efficacy.^[^ ^1^, ^2^, ^3^ ^]^ Moreover, the use of “active” drug delivery systems, in which the drug is released under defined external conditions (e.g., at a certain pH value or in the presence of a magnetic field) can achieve a targeted release at a desired location in the body. Among many other classes of porous materials, zeolites, crystalline inorganic materials with a tetrahedral framework structure, have received considerable attention as materials for drug delivery applications.^[^ ^2^, ^4^, ^5^ ^]^ In aluminosilicate zeolites, the tetrahedral framework sites are occupied by Si and Al atoms with a general framework composition Si_1‐x_Al_x_O_2_ (*x* ≤ 0.5).^[^ ^6^ ^]^ The Si/Al ratio determines the framework charge and consequently, the amount of positively charged species that are required for charge neutrality. Out of the variety of known zeolite frameworks types, zeolites having the FAU (faujasite) topology are particularly widely used in industrial processes, especially in catalysis and separation.^[^ ^7^ ^]^ Due to their good availability, large pore size, and the possibility to incorporate various species as extra‐framework cations, they have also been the focus of many drug delivery investigations. FAU‐type zeolites with Si/Al ratios below 1.5 are dubbed “zeolite X”. Zeolite X samples exchanged with different cations were investigated for the storage and delivery of the anticancer drug cyclophosphamide,^[^ ^8^ ^]^ the anti‐inflammatory and cytostatic 6‐mercaptopurine,^[^ ^9^ ^]^ the antiosteoporosis drugs risedronate and zoledronate,^[^ ^10^ ^]^ and the antibiotic ciprofloxacin^[^ ^11^ ^]^ among other species. More silicon‐rich FAU‐type zeolites are labelled “zeolite Y”, they were studied, for example, as host materials for the nonsteroidal anti‐inflammatory drug ibuprofen^[^ ^12^ ^]^ and for the antibiotics sulfadiazine^[^ ^13^ ^]^ and isoniazid.^[^ ^14^ ^]^ Looking beyond drug delivery, highly siliceous zeolite Y which possesses hydrophobic properties could find use as adsorbent for the removal of pharmaceuticals and other organic contaminants from wastewaters.^[^ ^15^, ^16^, ^17^, ^18^ ^]^

**Figure 1:**
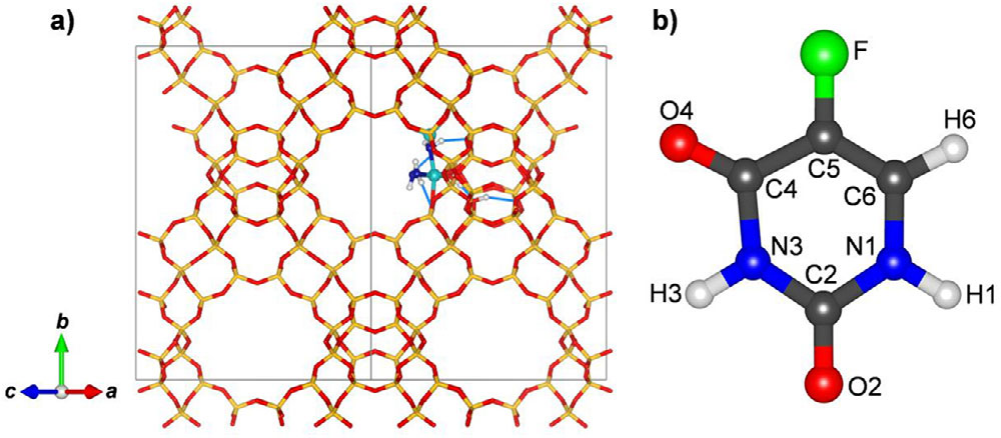
a) Unit cell of the FAU_fwAl_oct__2Al model. Si (yellow) and O_fw_ (red) atoms are shown in a stick representation. Al atoms (cyan) and H_2_O molecules (blue + white) are emphasized as balls. Hydrogen bonds are indicated as thin blue lines. b) Molecular structure and labelling scheme of the 5‐fluorouracil molecule.

5‐fluorouracil (FU) (C_4_H_3_FN_2_O_2_, CAS No. 51 − 21 − 8, Figure 1, abbreviated 5‐FU in the following) is a cytostatic drug that is used in the treatment of various types of cancer; it is typically administered intravenously. Due to its limited bioavailability and low absorption, high doses are required often resulting in severe side effects. Moreover, high dosages can promote the development of chemoresistance to 5‐FU, reducing its therapeutic efficacy.^[^ ^19^ ^]^ In order to mitigate these drawbacks, the potential applicability of various materials as 5‐FU carriers has been investigated.^[^ ^20^, ^21^ ^]^ The first investigation of 5‐FU storage and release using zeolites was published by Datt et al. in 2013, who compared three different samples of protonic zeolite Y with Si/Al ratios of 2.5, 15, and 30 (in protonic zeolites, protons bonded to framework oxygen atoms balance the framework charge).^[^ ^22^ ^]^ While they observed similar 5‐FU loadings on the order of 10 wt% for all three samples, the release behavior was marked differently: Whereas the two more Si‐rich samples released about 60% of the adsorbed 5‐FU in a few minutes, the most Al‐rich sample retained about 95%. On the basis of spectroscopic investigations (^27^Al NMR, infrared), it was inferred that 5‐FU forms strongly bound complexes with octahedrally coordinated aluminum (Al_oct_) sites. As these sites were most abundant in the most Al‐rich sample, it was concluded that the limited release from this sample was caused by a strong binding of 5‐FU to octahedral Al atoms. Octahedral Al sites, which show Lewis acidic properties, are often by default classified as “extra‐framework aluminum” [EFAl] sites, which are fully dislodged from the framework. However, it has been established that octahedral Al atoms can occur both as EFAl sites and as “framework‐associated” Al sites, which are only partially dislodged and retain some bonds to framework oxygen atoms.^[^ ^23^ ^]^ The relative amount of the two Al_oct_ species varies depending on the external conditions, with harsh conditions favoring EFAl formation, and their rigorous distinction with experimental methods is not trivial.^[^ ^24^ ^]^

Following the study by Datt et al., a number of other groups investigated 5‐FU adsorption in different zeolites including Na‐Y and K‐LTL,^[^ ^25^ ^]^ Na‐X and Na‐beta,^[^ ^26^ ^]^ Na‐Y, Na‐LTA, and NH_4_‐ZSM‐5,^[^ ^27^ ^]^ protonic zeolite beta,^[^ ^28^ ^]^ and Ag‐X.^[^ ^29^ ^]^ Cytotoxicity studies with human cancer cells that were performed in the context of some of these studies showed a notable increase in efficiency of zeolite‐encapsulated 5‐FU compared to the free drug.^[^ ^25^, ^26^, ^27^ ^]^ In one investigation, molecular dynamics (MD) simulations using classical force field parameters were used to study the diffusion of 5‐FU through the pores FAU‐ and BEA‐type zeolites.^[^ ^26^ ^]^ These simulations helped to rationalize the experimental observation of a slower 5‐FU release from zeolite beta as compared to Na‐X, as it was inferred that the tight fit of 5‐FU into the pores of zeolite beta gives rise to strong van der Waals (vdW) interactions.

A recent computational study investigated the adsorption of 5‐FU in protonic FAU‐type zeolites by means of dispersion‐corrected DFT calculations.^[^ ^30^ ^]^ In idealized models of protonic FAU, 5‐FU was found to interact strongly with the framework protons and simultaneous interaction with two framework protons resulted in a significant additional stabilization of the adsorption complex compared to a system containing only a single proton per twelve‐membered ring (12MR). In a water‐free system, the hydrogen bonds between 5‐FU oxygen atoms and framework protons remained stable in DFT‐based AIMD simulations (*T* = 298 K). When adding water molecules, however, these bonds were broken within a few picoseconds, the adsorbed 5‐FU molecule was displaced into the pore and the framework was deprotonated by the cluster of water molecules. Based on that observation, it was concluded that the exposure to water could be a suitable pathway to trigger 5‐FU release from protonic zeolites.

Although that previous computational study investigated the same zeolite framework and similar Si/Al ratios as the earlier experimental study by Datt et al.,^[^ ^22^ ^]^ some important disagreements were noted: First, the DFT‐calculated IR spectra of 5‐FU‐loaded FAU showed poor agreement with experimental data. Second, all three samples studied experimentally showed only partial 5‐FU release (and in the case of the most Al‐rich sample almost no release at all), while the computations indicated a fast, complete release of the drug from the zeolite host. This discrepancy was explained with the dominant adsorption of 5‐FU at Lewis acidic Al_oct_ sites in the experimental samples, whereas the DFT calculations considered only Brønsted acidic framework protons. The present work addresses this issue by explicitly considering the interaction of 5‐FU with octahedral Al sites in FAU‐type zeolites.

The experimental detection of octahedral Al atoms in zeolites has been established for decades, as they give rise to a characteristic signal at about 0 ppm in solid‐state ^27^Al NMR spectroscopy (tetrahedral Al atoms: 50–60 ppm, reference: aqueous solution of Al(NO_3_)_3_).^[^ ^6^ ^]^ In contrast, the atomistic modelling of such sites is far from trivial and different types of octahedral aluminum species have been proposed in prior computational work.^[^ ^31^, ^32^, ^33^, ^34^, ^35^, ^36^ ^]^ Recently, Mancuso and van Speybroeck employed a combination of DFT calculations and DFT‐based AIMD simulations to investigate different types of framework‐associated and extra‐framework Al sites in H‐ZSM‐5, considering realistic conditions for methanol‐to‐hydrocarbon conversion reactions.^[^ ^36^ ^]^ They predicted two types of sites to be stable under operating conditions, one being a framework‐associated [Al(OH)_2_]^+^ species and the other one being a [Al(OH)_2_(H_2_O)_2_]^+^ entity that exists as a “pore guest” that is not bonded to the framework. While both of these environments consisted of tetrahedrally coordinated Al atoms, they inferred that the presence of additional H_2_O molecules in the system could shift the equilibrium towards higher Al coordination numbers. The role of water was also investigated in a combined experimental and computational study of CHA‐type zeolites by Jin et al.^[^ ^35^ ^]^ They identified two key factors that stabilize framework‐associated octahedral Al sites. First, the proximity of framework protons (Brønsted acid sites) and second, the presence of several additional water molecules in the vicinity (“wet” conditions). The zeolite model containing an octahedral Al atom used in the present work was constructed in analogy to the most stable model developed by Jin et al., in which the framework‐associated Al_oct_ site is stabilized by two framework protons in the vicinity. This model was chosen because it seems reasonable to assume that octahedral Al atoms are dominantly present as framework‐associated species in hydrated samples that have not undergone harsh treatment.^[^ ^24^ ^]^ Throughout this article, FAU models containing framework‐associated Al_oct_ sites are labelled with the designator FAU_fwAl_oct_.

The remainder of this article is organized as follows: After describing the structure models and computational details, the stability of a framework‐associated octahedral Al environment in the absence and presence of additional H_2_O molecules (that are not coordinated to the Al_oct_ atom) is investigated with AIMD simulations. Following this, DFT optimizations of models where one 5‐FU molecule is coordinated to the Al_oct_ site are performed, allowing the calculation of adsorption energies and an analysis of the local environment of 5‐FU. In order to establish a link to experimental findings, IR spectra and ^27^Al NMR isotropic chemical shifts are computed for selected models. Finally, AIMD simulations of models with Al‐coordinated 5‐FU in the absence and presence of a hydration shell are carried out, allowing conclusions on the stability of such complexes under conditions relevant for drug delivery applications.

## 2 Computational Details

### 2.1 Structure Models: FAU With an Octahedral Al Site

A DFT optimized structure of all‐silica FAU constituted the starting point for the construction of models containing a framework‐associated octahedral Al atom. The unit cell parameters were fixed to the cell parameters of all‐silica FAU obtained in previous work (*a* = 24.227 Å).^[^ ^30^, ^37^, ^38^ ^]^ The model in which the Al_oct_ atom is located in the vicinity of a tetrahedral Al (Al_tet_) atom, dubbed FAU_fwAl_oct__2Al in the following, was constructed along the lines of the work of Jin et al.^[^ ^35^ ^]^ The octahedral Al atom is coordinated to three framework oxygen atoms (O_fw_) and three H_2_O molecules (unit cell composition: Si_190_Al_2_O_382_(OH)_2_(H_2_O)_3_, Figure 1). The tetrahedral Al atom is in a next‐nearest neighbour position of the Al_oct_ site. Charge balance is ensured through two framework protons, one of them saturating the “dangling” oxygen atom that arises from the breakage of one Si−O_fw_−Al link around the Al_oct_ atom, and the other one being bonded to an O_fw_ atom of a Si−O_fw_−Al_oct_ link. It is worth pointing out that the Si/Al ratio of the model is relatively high (Si/Al = 95), higher than those of the FAU samples investigated experimentally.^[^ ^22^ ^]^ However, since the present study focuses on the interaction of 5‐FU with a single octahedrally coordinated Al atom, it was not the goal to reproduce the Si/Al ratio of these samples. Such an endeavor would require further assumptions about the location and coordination of other Al atoms and the location of charge‐balancing species, thus complicating the model building while likely having little impact on the interaction with 5‐FU on a local level. Based on the FAU_fwAl_oct__2Al model, three models having five‐coordinated Al sites were constructed by removing one of the three water molecules. In addition, another model dubbed FAU_fwAl_oct__1Al was considered initially. In that model, there is no tetrahedral Al site and consequently only one framework proton.

In order to investigate the stabilization of the octahedral Al site through water molecules, six water molecules were added to the FAU_fwAl_oct__2Al model. This was done by means of auxiliary force field simulations, which are described in more detail in sections S1.1 and S1.2 (Supporting Information).^[^ ^39^, ^40^, ^41^ ^]^ Five low‐energy snapshots were extracted from the simulation results and optimized with DFT. The structure having the lowest energy was then taken as starting point for the AIMD simulations.

### 2.2 Structure Models: FAU with an Octahedral Al Site and Adsorbed 5‐FU

The structure of 5‐FU was taken from the PubChem database.^[^ ^42^ ^]^ The labelling scheme of the atoms shown in Figure 1 follows earlier work.^[^ ^30^, ^43^ ^]^ A total of six models of FAU_fwAl_oct__2Al with the 5‐FU molecule replacing one of the Al‐coordinated H_2_O molecules were constructed, considering a replacement of each of the three H_2_O molecules (labelled H_2_O(1), H_2_O(2), H_2_O(3)) and a coordination of 5‐FU via the O2 or O4 oxygen atoms (composition: Si_190_Al_2_O_382_(OH)_2_(H_2_O)_2_(5‐FU)). Out of these six models, the two having the lowest energy according to DFT optimizations were subjected to further AIMD simulations. Moreover, the effect of the presence of water molecules was evaluated by adding 14 water molecules, again using auxiliary force field simulations. For these hydrated 5‐FU@FAU_fwAl_oct__2Al models, six separate AIMD simulations were performed, considering two different 5‐FU positions and, for each, three different snapshots from the force field simulations of water adsorption. The total number of water molecules in these systems is the same as in an earlier study, where 16 H_2_O molecules were added to 5‐FU@H‐FAU models (in keeping with prior work, H‐FAU represents models of protonic FAU that contain only tetrahedral Al atoms and framework protons).^[^ ^30^ ^]^

### 2.3 Density Functional Theory Calculations and AIMD Simulations

All DFT and AIMD simulations used the Quickstep code that is integrated in the CP2K software.^[^ ^44^, ^45^ ^]^ This code uses the Gaussian and plane wave method (GPW) employing an auxiliary plane wave basis set within a Gaussian orbital scheme. All calculations used “molecularly optimized” Gaussian basis sets from the work of Vandevondele and Hutter^[^ ^46^ ^]^ employing basis sets of double‐zeta quality (DZVP‐MOLOPT‐SR) for AIMD simulations and triple‐zeta basis sets (TZVP‐MOLOPT and TZVP‐MOLOPT‐SR) for DFT optimizations and calculations of vibrational spectra. To model the core electrons, Gaussian‐type Goedeker–Teter–Hutter (GTH) pseudopotentials derived by Krack were used.^[^ ^47^ ^]^ As in the previous study of 5‐FU adsorption in H‐FAU,^[^ ^30^ ^]^ the dispersion‐corrected rev‐vdW‐DF2 functional was used,^[^ ^48^ ^]^ which was found to perform well in a benchmarking of DFT adsorption energies against results from high‐level coupled‐cluster calculations.^[^ ^49^ ^]^

Structure optimizations and vibrational calculations used triple‐zeta basis sets and a cutoff energy of 900 Ry. Fixing the unit cell parameters, all atomic coordinates were optimized using a BFGS optimizer, enforcing the following convergence criteria: Maximal gradient: 5·10^−6^ Ha bohr^−1^; maximal displacement: 10^−5^ bohr (1 Ha = 2625.5 kJ mol^−1^, 1 bohr = 0.52918 Å). All structure visualizations were prepared using VESTA.^[^ ^50^ ^]^ Calculations of IR spectra of adsorbed 5‐FU used the partial Hessian approach, including only displacements of 5‐FU and the closest framework atoms. IR spectra were plotted using MOLDEN, employing a Lorentzian broadening with a full width at half maximum of 10 cm^−1^.^[^ ^51^, ^52^ ^]^

AIMD simulations used double‐zeta basis sets and a cutoff energy of 600 Ry. They were carried out in the canonical ensemble (constant *N*, *V*, and *T*) using a time step of 0.5 fs and a Nosé‐Hoover thermostat with a time constant of 50 fs.^[^ ^53^, ^54^ ^]^ Different temperatures (298, 373, and 473 K) and different durations (from 12.5 ps up to 25 ps, i.e., between 25000 and 50000 steps) were considered in different simulations as detailed in the results section. From the AIMD trajectories, snapshots were extracted every 2500 steps (= every 1.25 ps). Each of these snapshots was fully optimized as described above, thereby allowing to probe several local minima.

Finally, overlap (bond) populations based on a Mulliken population analysis as well as ^27^Al NMR isotropic chemical shifts were computed for selected DFT‐optimized structures. These calculations used the CASTEP plane wave DFT code,^[^ ^55^, ^56^ ^]^ employing a projection of plane wave states onto a localized basis set for the population analysis^[^ ^57^ ^]^ and the gauge‐including projector augmented‐wave (GIPAW) method^[^ ^58^, ^59^ ^]^ to compute NMR parameters. The PBE exchange‐correlation functional was used.^[^ ^60^ ^]^ Isotropic chemical shielding were converted into chemical shifts using an equation proposed in a recent DFT study of zeolites, which employed a very similar computational approach.^[^ ^61^ ^]^ Further details are provided in section S1.3.

## 3 Results and Discussion

### 3.1 Stability of the Octahedral Al Environment

**Figure 2:**
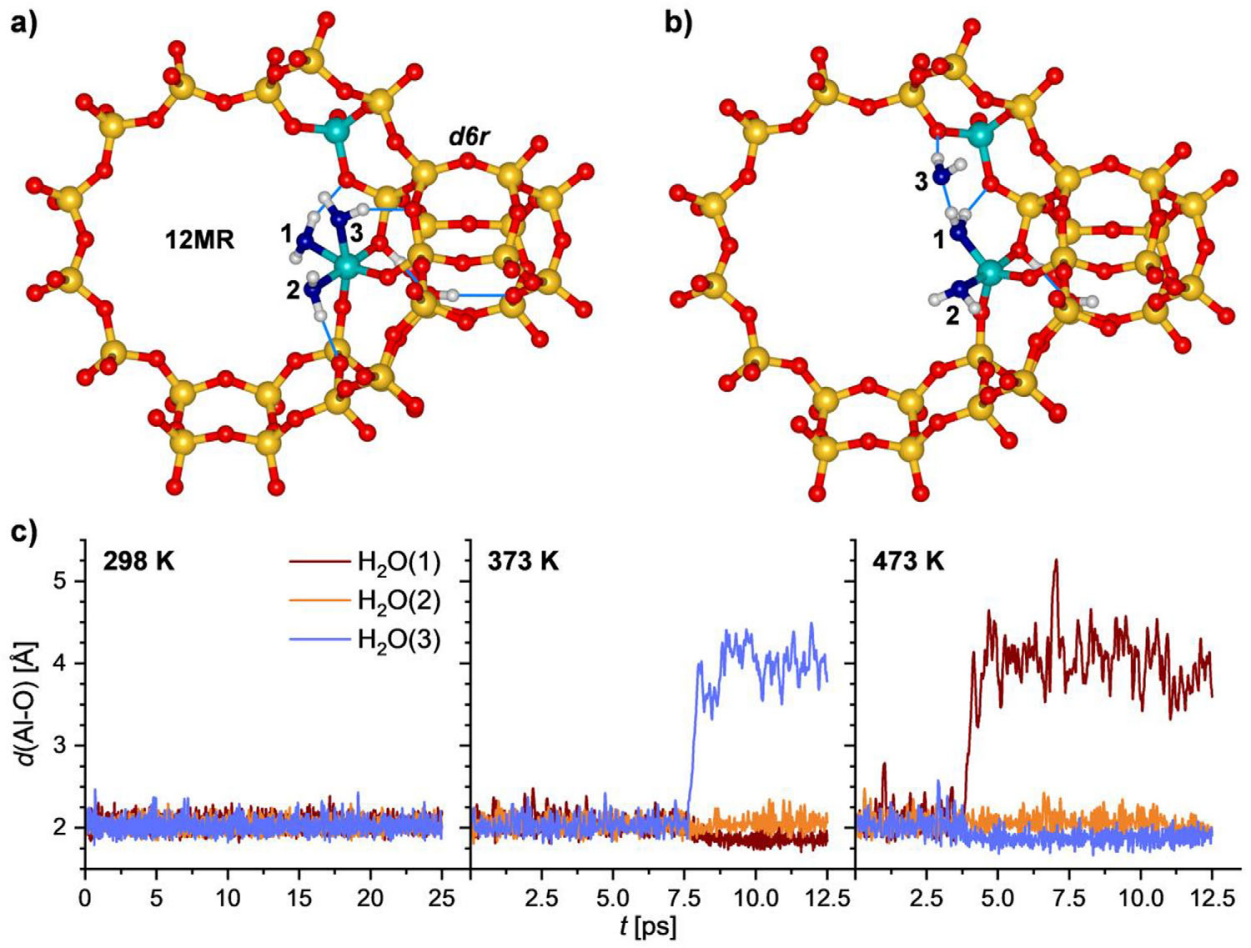
a) Low‐energy structure of intact framework‐associated Al_oct_ site (optimized AIMD snapshot from 298 K trajectory). O atoms of H_2_O molecules are shown in blue. As in the following figures, only the direct environment of the Al_oct_ site is shown for clarity. b) Low‐energy structure after dissociation of one Al─O(H_2_O) bond (optimized AIMD snapshot from 373 K trajectory). c) Time evolution of Al─O(H_2_O) distances during AIMD simulations.

After constructing a FAU_fwAl_oct__2Al model and a preliminary DFT optimization, AIMD simulations for 298 K (duration: 25 ps) and for 373 K and 473 K (12.5 ps) were run to probe the stability of this model under conditions of interest. Figure 2c shows the evolution of the three Al−O(H_2_O) distances during the AIMD simulations. At 298 K, the distances remain in an interval between about 1.8 and 2.5 Å, and the octahedral environment remains intact over the course of the 25 ps simulation. Figure 2a shows the lowest‐energy structure obtained in a series of optimizations of snapshots from the 298 K trajectory. It is only 0.3 kJ mol^−1^ (per unit cell) lower in energy than the initial DFT‐optimized FAU_fwAl_oct__2Al model. The figure also introduces the labelling of the three H_2_O molecules that are (at least initially) coordinated to the Al_oct_ atom: The H_2_O(1) molecule is located approximately in the plane of the 12MR forming a hydrogen bond to an O_fw_ atom that is bonded to the tetrahedral Al site. H_2_O(2) lies in the same equatorial plane (defined with respect to the neighboring double six‐membered ring – *d6r*) as H2O(1), but lies above an adjacent 6MR. H_2_O(3) occupies an axial position and is associated with one of the 4MRs belonging to the *d6r* unit.

At 373 K and 473 K, the evolution of the Al−O(H_2_O) distances over time clearly show that one Al─O bond is broken, resulting in an increase of one of the distances to values above 4 Å. The energies of optimized AIMD snapshots are shown in Figure S1b, which shows unambiguously that the dissociated state with a trigonal‐bipyramidal Al atom is energetically favored over the initial model with octahedrally coordinated Al. The most stable optimized configuration obtained from the 373 K trajectory is shown in Figure 2b. In this configuration, the H_2_O(3) molecule is no longer coordinated to the Al atom, but has moved towards one O_fw_ atom that neighbors the Al_tet_ atom, simultaneously acting as hydrogen bond donor (to O_fw_) and acceptor (from H_2_O(1)). In the simulation run for 473 K, the bond from Al_oct_ to H_2_O(3) instead of H2O(1) is broken, with the detached H_2_O molecule forming a hydrogen bond to an O_fw_ atom belonging to a Si─O_fw_─ Si link. Altogether, these results clearly indicate that the octahedral Al environment in FAU_fwAl_oct__2Al is inherently unstable under “nonhydrated” conditions (i.e., in the absence of noncoordinated H_2_O molecules). Before investigating the role of additional water molecules, it is worth mentioning that a FAU_fwAl_oct__1Al model (without Al_tet_ atom) dissociates in AIMD simulations within a few picoseconds, even at 298 K, ultimately forming a tetrahedral framework‐associated Al site (Al(O_fw_)_3_(H_2_O)). Thus, proximity of a framework Al atom and an associated framework proton appear to be of crucial importance to stabilize a framework‐associated octahedral Al environment, in line with the findings of Jin et al.^[^ ^35^ ^]^

**Figure 3:**
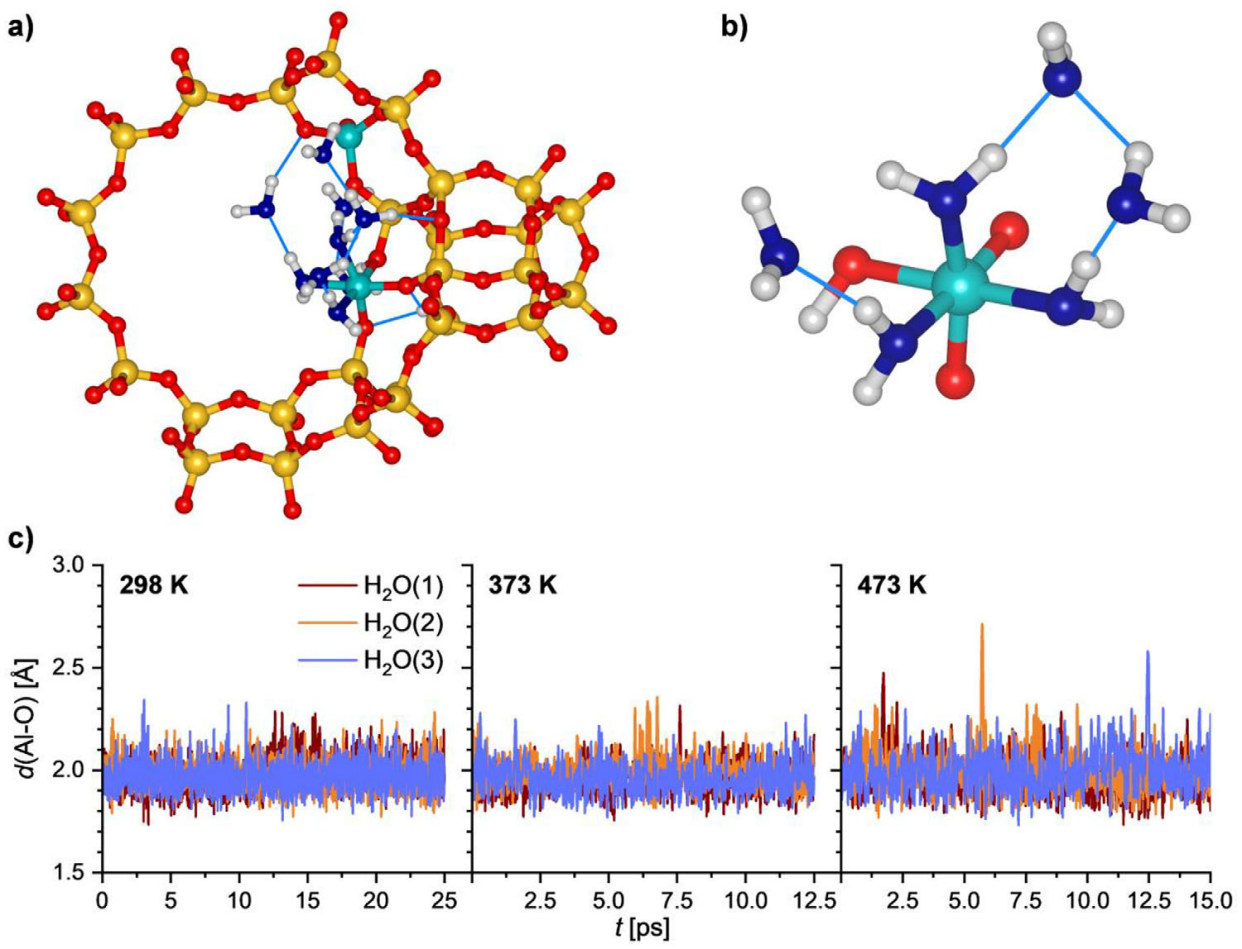
a) Low‐energy structure of FAU_fwAl_oct__2Al in model containing six additional H_2_O molecules (optimized AIMD snapshot from 298 K trajectory). b) Local environment of the Al_oct_ atom in the same structure. c) Time evolution of Al─O(H_2_O) distances during AIMD simulations considering only coordinated H_2_O molecules.

Figure 3c shows the time evolution of the Al─O(H_2_O) distances for FAU_fwAl_oct__2Al models containing six additional H_2_O molecules. It is clearly visible that no breaking of any Al─O(H_2_O) bond occurs. Even though there are some “spikes” in the 473 K trajectory with individual distances exceeding 2.5 Å for very short time intervals, the H_2_O molecules always return to the Al atom. The environment of the Al_oct_ atom is visualized in Figure 3a,b with the second panel emphasizing the local environment of the Al‐coordinated H_2_O molecules. Each of the three molecules forms a hydrogen bond to another, noncoordinated H_2_O molecule and it seems safe to conclude that the availability of these additional molecules is a crucial factor in stabilizing the octahedral environment. However, since the calculations considered only a fixed number of water molecules (six, i.e., two noncoordinated per coordinated H_2_O molecule), no conclusions regarding the minimum number of H_2_O molecules required to stabilize the octahedral Al environment site can be drawn.

### 3.2 Adsorption of 5‐FU at Octahedral Al Sites: Adsorption Energies and Structures

**Figure 4:**
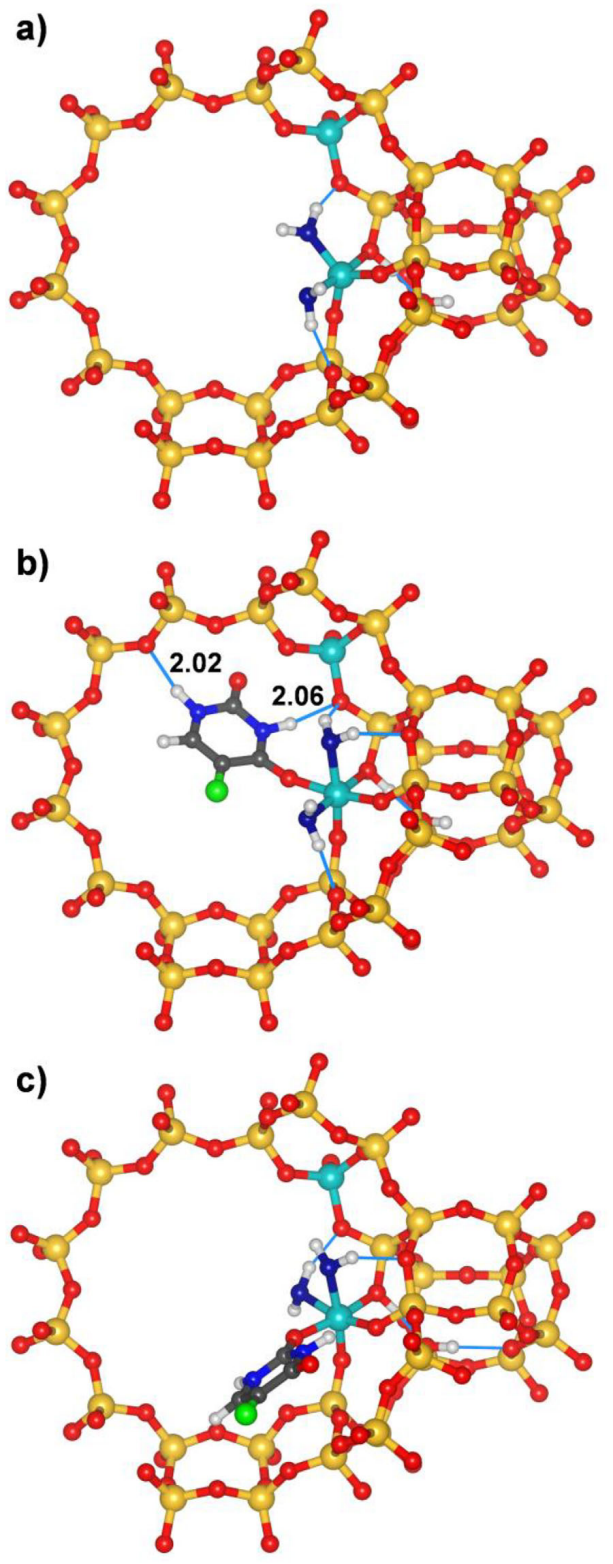
a) Reference structure with trigonal‐bipyramidal Al atom (FAU_fwAl_tbp__2Al). b) and c) Lowest‐energy structures with Al‐coordinated 5‐FU, configurations labelled b) 5‐FU_1_O4 and c) 5‐FU_2_O2.

Even though the above calculations have established that the octahedral Al environment requires additional H_2_O molecules for stabilization, the calculations including Al‐coordinated 5‐FU considered in the first place only the “bare” Al_oct_ site without additional water molecules. This simplification is primarily motivated by the complications that would arise from an inclusion of noncoordinated water molecules in the calculation of the adsorption energy: In such a system, there is no straightforward way to disentangle different contributions to the total energy (framework–water, framework–5‐FU, 5‐FU–H_2_O, H_2_O–H_2_O), precluding an unambiguous decomposition that would allow to compute 5‐FU adsorption energies. As detailed in 2.2, a total of six 5‐FU@FAU_fwAl_oct__2Al configurations were optimized, which differ in the water molecule that was replaced by 5‐FU and the coordination mode of 5‐FU (“O2‐on” versus “O4‐on”). Two different possibilities to analyze the total energy were considered, which result in different absolute values but identical trends. The first possibility takes the lowest‐energy model having a five‐coordinated (trigonal‐bipyramidal) Al atom (labelled FAU_fwAl_tbp__2Al, Figure 4a) as reference structure and calculates the adsorption energy *E*_ads_ as follows:

(1) $$\begin{array}{ccc} E_{\mathrm{ads}} & = & E_{\mathrm{DFT}}\left(5\mathrm{FU}@\mathrm{FAU}\_\mathrm{fwA}l_{\mathrm{oct}}\_2\mathrm{Al}\right)\\ & & -\;E_{\mathrm{DFT}}\left(\mathrm{FAU}\_\mathrm{fwA}l_{\mathrm{tbp}}\_2\mathrm{Al}\right)-\;E_{\mathrm{DFT}}\left(5\mathrm{FU}\right) \end{array}$$

The second possibility takes the lowest‐energy structure of FAU_fwAl_oct__2Al as reference and calculates the “replacement energy” *E*_repl_ associated with a replacement of H_2_O by 5‐FU:

(2) $$\begin{array}{ccc} E_{\mathrm{repl}} & = & E_{\mathrm{DFT}}\left(5\mathrm{FU}@\mathrm{FAU}\_\mathrm{fwA}l_{\mathrm{oct}}\_2\mathrm{Al}\right)-\;E_{\mathrm{DFT}}\left(\mathrm{FAU}\_\mathrm{fwA}l_{\mathrm{oct}}\_2\mathrm{Al}\right)\\ & & +\;E_{\mathrm{DFT}}\left(H_{2}O\right)-\;E_{\mathrm{DFT}}\left(5\mathrm{FU}\right) \end{array}$$

For all six configurations, the adsorption and replacement energy values, interatomic distances between the Al_oct_ atom and the coordinated O atom of 5‐FU, as well as atoms participating in hydrogen bonds are listed in Table 1. Despite different 5‐FU positions, coordination modes, and hydrogen bonding patterns five out of six configurations are close in energy, falling within about 6 kJ mol^−1^. Among them, the configurations indexed 1_O4 and 2_O2 have the lowest adsorption energies and will therefore be in the focus of the following discussion.

**Table 1:** Adsorption energy and replacement energy (see Equation (1) and (2)) of 5‐FU@FAU_fwAl_oct__2Al models, *d*(Al─O_5‐FU_) bond distances, and list of hydrogen bonds.

Index	Replaced Molecule	5‐FU Coordination	*E*_ads_ [kJ mol^−1^]	*E*_repl_ [kJ mol^−1^]	*d*(Al − O_5‐FU_) [Å]	Hydrogen Bond(s)
1_O2	H_2_O(1)	O2‐on	−106.6	−39.2	1.932	N3─H3···O_fw_
**1_O4**	H_2_O(1)	O4‐on	**−118.7**	−51.2	1.914	N1─H1···O_fw_ N3─H3···O_fw_
**2_O2**	H_2_O(2)	O2‐on	**−117.2**	−49.8	1.919	None
2_O4	H_2_O(2)	O4‐on	−114.3	−46.8	1.927	None
3_O2	H_2_O(3)	O2‐on	−112.6	−45.2	1.963	N1─H1···O_fw_
3_O4	H_2_O(3)	O4‐on	−114.6	−47.1	1.955	N3─H3···O_fw_

To begin with, it is useful to compare the adsorption energy of 5‐FU@FAU_fwAl_oct__2Al to values computed for other FAU models in a previous computational study using a largely analogous setup:^[^ ^30^ ^]^ In that work, an *E*_ads_ value of −73 kJ mol^−1^ was obtained for an all‐silica FAU model, whereas the adsorption energy for 5‐FU interacting with a single framework proton (H‐FAU_1H) amounted to −144 kJ mol^−1^ (a re‐calculation in the context of the present work that used TZVP rather than DZVP basis sets for the entire optimization resulted in a marginally changed value of −146 kJ mol^−1^). Thus, the interaction of 5‐FU with a Lewis acidic Al_oct_ site is considerably stronger than for an all‐silica zeolite, but significantly weaker than the interaction with a Brønsted acidic site in a protonic zeolite. The negative replacement energies on the order of −50 kJ mol^−1^ indicate that the replacement of one H_2_O molecule by one 5‐FU molecule in the environment of the Al atom is energetically favored. However, it has to be noted in this context that Al‐coordinated H_2_O molecules are stabilized by additional water molecules, as demonstrated above. Due to the size of the 5‐FU molecule, it can be anticipated that a displacement of several H_2_O molecules would be required to enable a coordination of 5‐FU to the Al_oct_ atom. The calculations presented here do not take this into account, and therefore do not allow for direct predictions under what conditions a replacement of H_2_O by 5‐FU would take place.

Figure 4b,c visualize the two lowest‐energy 5‐FU@FAU_fwAl_oct__2Al configurations. In the first configuration, dubbed 5‐FU_1_O4, the 5‐FU molecule lies essentially in the plane of the 12MR, forming relatively short hydrogen bonds to O_fw_ atoms lining the 12MR through both ─NH groups. In the second configuration, 5‐FU_2_O2, the adsorbed 5‐FU molecule has a completely different orientation and local environment, lying flat above two edge‐sharing 4MRs that form the wall of the supercage. There are no hydrogen bonds, but numerous C_5‐FU–_O_fw_ and N_5‐FU–_O_fw_ contacts in the distance range from 3.2 to 3.6 Å, thus being close to the sum of vdW radii (r_vdW_(C) + r_vdW_(O) = 3.27 Å; r_vdW_(N) + r_vdW_(O) = 3.43 Å^[^ ^62^ ^]^). While a direct separation of the dispersion contribution from the total adsorption energy is not possible when using a nonlocal vdW‐DF functional, it can still be inferred from these observations that different interaction types contribute to the stabilization of the two lowest‐energy configurations: While the Al_oct _− O_5‐FU_ bond is expected to be similar in strength for both configurations, the additional stabilization of the specific position and orientation of the 5‐FU molecule stems from hydrogen bonds in one case (1_O4) and from dispersion interactions in the other case (2_O2). Incidentally, the contributions of the different interaction types result in a near‐identical total interaction strength, and it can hence be assumed that both bonding scenarios can co‐exist in real systems.

Further insights into the nature of the bonds between the Al_oct_ atom and coordinated molecules can be obtained from a Mulliken population analysis. Table S4a shows the Mulliken charges of the Al atoms computed for selected structures, including the FAU_fwAl_oct__2Al model and the two lowest‐energy 5‐FU@FAU_fwAl_oct__2Al configurations. The overlap populations are tabulated in Tables S4b–S4g. While the Mulliken charge of the octahedral Al atom remains practically unchanged when replacing one H_2_O molecule by 5‐FU, the overlap populations provide more useful insights: For intra‐framework Al─O bonds, overlap populations are on the order of 0.4 |*e*|, whereas Al_oct─_O(H_2_O) bonds are usually characterized by an overlap population of about 0.2 |*e*|. This indicates that the intra‐framework bonds have a significant covalent component, whereas Al_oct─_O(H_2_O) interactions are dominantly electrostatic.^[^ ^57^ ^]^ The overlap populations of the Al_oct_−O(5‐FU) bond in the two lowest‐energy structures with coordinated 5‐FU molecules amount to 0.27/0.26 |*e*|, pointing to a nonnegligible increase in covalency with respect to Al_oct_─O(H_2_O) bonds. This increased covalent component serves to explain why a replacement of H_2_O by 5‐FU in the first coordination shell of the Al_oct_ atom is energetically favored, and why the Al_oct_─O(5‐FU) bonds are relatively stable, as indicated by experimental observations and further corroborated by the AIMD simulations reported in 3.4. As an additional point, one may also note that the overlap populations of the Al_oct_─O(H_2_O) bonds in the FAU_fwAl_oct__1Al model are unusually small, falling between 0.14 and 0.17 |*e*|. Apparently, the absence of a Brønsted acid site in the direct vicinity of the Al_oct_ site results in a weaker interaction with the coordinated water molecules. This weak binding is responsible for the fast dissociation of the octahedral coordination environment of FAU_fwAl_oct__1Al that occurs in the AIMD simulations.

### 3.3 Adsorption of 5‐FU at Octahedral Al Sites: IR and NMR Spectra

**Figure 5:**
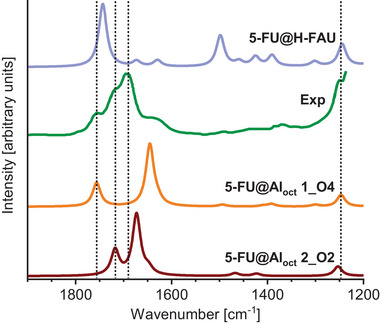
IR spectra of 5‐FU‐containing FAU. Green: Experimental spectrum of most Si‐rich sample studied in ref. [22]. Blue: DFT‐calculated spectrum of 5‐FU adsorbed at framework proton of H‐FAU_1H. Orange and dark red: DFT‐calculated spectra of low‐energy 5‐FU@FAU_fwAl_oct__2Al models. Dashed lines indicate four vibrational bands that were assigned experimentally (see text).

In their experimental study, Datt et al. presented IR spectra of three FAU samples loaded with 5‐FU.^[^ ^22^ ^]^ Since spectra of zeolites with different Si/Al ratios were very similar, Figure 5 visualizes the IR spectrum of the most Si‐rich sample (dubbed HY‐60 in that work, Si/Al ratio = 30). Datt et all. identified four bands in the wavenumber range from 1800 to 1200 cm^−1^, which were attributed to C═O stretching (*ν*(C2═O2) at 1756 cm^−1^, *ν*(C4═O4) at 1717 cm^−1^), C═C stretching (*ν*(C5═C6) at 1690 cm^−1^), and C−F stretching (*ν*(C5 − F) at 1247 cm^−1^). These experimental values are shown as vertical lines in Figure 5. The figure also shows DFT‐calculated IR spectra obtained for the two lowest‐energy 5‐FU@FAU_fwAl_oct__2Al configurations discussed above and to a spectrum computed for a system where 5‐FU interacts with the framework proton of a protonic zeolite (5‐FU@H‐FAU_1H, newly optimized in the present work). Starting with the spectrum of 5‐FU@H‐FAU_1H, it is apparent that several features do not match the experimental observations: Most notably, there is only one major C═O stretching vibration at 1743 cm^−1^. The second C ═O stretching band is red‐shifted to 1673 cm^−1^ due to the formation of a C4═O4···H_fw_ hydrogen bond and its intensity is reduced considerably. The formation of this hydrogen bond also gives rise to a relatively intense band related to O_fw–_H_fw_ ···O4 angle bending at about 1500 cm^−1^, which is not observed in the experimental spectrum at all.

When turning to the calculated spectra of the two 5‐FAU@FAU_fwAl_oct__2Al systems, both common features and differences are apparent, especially in the wavenumber range of 1750–1600 cm^−1^. Both spectra show a moderately intense band at higher wavenumbers, which stems from C═O stretching vibrations associated with the 5‐FU oxygen atom that is not bonded to the Al atom, and a more intense band at lower wavenumbers that is associated with vibrations of the O atom that is coordinated to Al_oct_ atom. However, the separation between the two bands is rather different, amounting to 111 cm^−1^ for configuration 1_O4, but only 45 cm^−1^ for 2_O2. In both cases, the *ν*(C5═C6) vibration makes only a slight contribution to the spectrum, appearing as a shoulder on the low wavenumber side of the intense band. With the exception of the *ν*(C5–F) band, all bands below 1600 cm^−1^ are very weak, agreeing with the lack of distinctive bands in the experimental spectrum in this wavenumber range. Altogether, neither of the two individual spectra shows a one‐to‐one correspondence with the experimental spectrum. Based on the good agreement of individual signals in the wavenumber range of 1750–1600 cm^−1^, it appears, however, plausible to assume that the experimental spectrum arises from vibrations of Al‐coordinated 5‐FU molecules in different local environments. While only one type of framework‐associated Al_oct_ site and two positions and orientations of 5‐FU were considered here, a real sample is likely to possess a variety of different environments of octahedral Al atoms (different parts of the pore, different degrees of association with the framework, etc.), which may in turn favor different coordination modes of 5‐FU. For illustrative purposes, composite IR spectra were prepared in which the relative contribution of the two configurations was varied in a certain range. As shown in Figure S6, IR spectra assuming a contribution of about 80% or 90% from the 2_O2 configuration and a 20% or 10% contribution from the 1_O4 configuration reproduce all key features of the experimental spectrum in the wavenumber range of 1750–1600 cm^−1^ even without applying any particular adjustment of the full width at half maximum (arbitrarily chosen as 10 cm^−1^).

Datt et al. also presented ^27^Al NMR spectra of their zeolite samples before and after the adsorption of 5‐FU.^[^ ^22^ ^]^ Calculated ^27^Al NMR chemical shifts are compiled in Table S5 for several of the FAU models considered here. For tetrahedral Al atoms, chemical shifts on the order of 56–59 ppm are predicted and there is no evident effect of the presence of 5‐FU on the position in agreement with experiment (∼60 ppm). For the octahedral Al atoms, a chemical shift of 1 to 3 ppm is predicted for systems without 5‐FU. This shift moves to about −3 to −8 ppm in systems where 5‐FU is coordinated to the Al atom. The experimental chemical shift of about −2 ppm seems to be less affected by 5‐FU adsorption, however, the considerable broadening of the corresponding peak in the two more Si‐rich samples renders it difficult to determine the chemical shift precisely. This broadening may at least partially be due to the co‐existence of different local environments of 5‐FU in real samples. Calculations for systems with trigonal‐bipyramidal Al atoms in which one of Al−O(H_2_O) bonds is broken, deliver chemical shifts in the range of 21–28 ppm. The absence of such peaks in the experimental NMR spectra indicates that five‐coordinated Al atoms had only limited abundance in the 5‐FU‐containing samples investigated by Datt et al.

### 3.4 Adsorption of 5‐FU at Octahedral Al Sites: Influence of Temperature and Co‐Adsorbed Water Molecules

**Figure 6:**
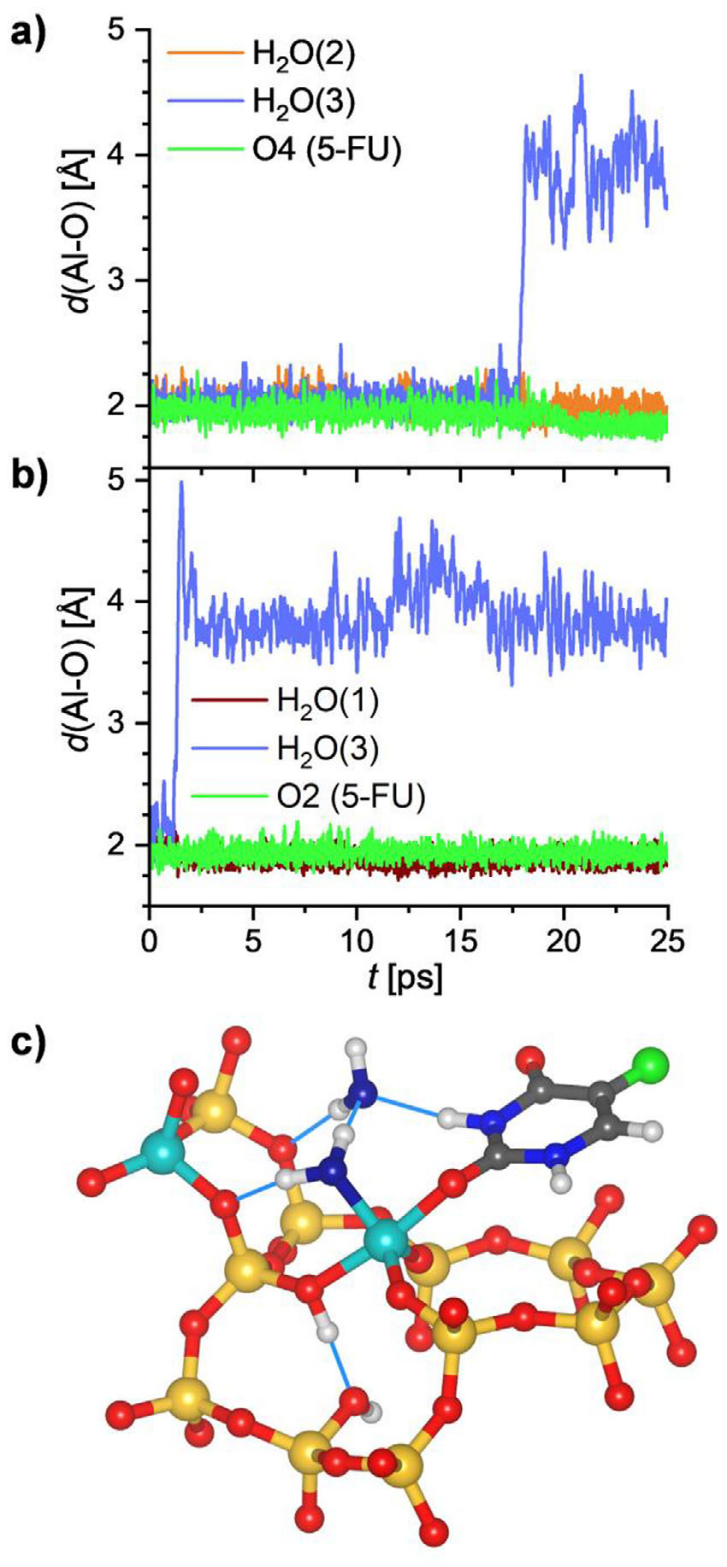
a) and b) Time evolution of Al─O distances during AIMD simulations of 5‐FU@FAU_fwAl_oct__2Al (*T* = 298 K), configurations 1_O4 a) and 2_O2 b). c) Low‐energy structure of 2_O2 configuration after dissociation of the Al─O(H_2_O) bond (optimized snapshot from AIMD trajectory). To emphasize relevant features, a different projection than in the preceding figures is used.

In order to probe the stability of the Al‐coordinated 5‐FU molecules at finite temperature, AIMD simulations were performed for a temperature of 298 K, starting from the two lowest‐energy configurations visualized in Figure 4. Figure 6 shows the evolution of the Al−O distances during the simulation time of 25 ps for the three Al‐coordinated species (5‐FU and two H_2_O molecules). It is evident that one of the Al−O(H_2_O) bonds dissociates during the simulation, in analogy to the observations made above for the FAU_fwAl_oct__2Al system at elevated temperatures in the absence of additional noncoordinated H_2_O molecules. A re‐optimization of snapshots shows a further lowering of the total energy by up to 26 kJ mol^−1^ after breaking of the bond in the 2_O2 configuration, whereas the total energy of the 1_O4 configuration remains rather close to its initial value (Figure S3). The energetically most favorable arrangement obtained from these optimizations is visualized in Figure 6c. In this structure, the water molecule that has been removed from the framework‐associated Al atom acts as hydrogen bond acceptor for the other H_2_O molecule and for 5‐FU as well as forming a hydrogen bond to the framework. The fact that no breaking of the Al−O(5‐FU) bond is observed in either of the simulations indicates that Al‐coordinated 5‐FU is more stable than Al‐coordinated H_2_O, in line with the negative replacement energies reported in Table 1 and the increased covalent character of the bond discussed above.

The impact of the presence of noncoordinated water molecules was evaluated by means of AIMD simulations including additional H_2_O molecules for the two 5‐FU@FAU_fwAl_oct__2Al models considered above. For this purpose, 14 H_2_O molecules were added to the system in preliminary GCMC simulations and for each configuration, three snapshots were taken as starting points for separate AIMD simulations. The evolution of the Al−O distances presented in Figure S4 for all six trajectories clearly demonstrates that no breaking of any Al−O bond occurs during these simulations. Apparently, noncoordinated water molecules have a stabilizing effect on the octahedral Al environment, regardless of whether the Al atom is bonded only to H_2_O molecules (as investigated in 3.1) or to both H_2_O and 5‐FU. A representative DFT‐optimized snapshot (Figure S5) shows the extended network of hydrogen bonds between coordinated and noncoordinated H_2_O molecules in which 5‐FU also participates, simultaneously acting as hydrogen bond donor and acceptor.

## 4 Conclusions

The DFT calculations and AIMD simulations performed in this work showed that 5‐FU can form a stable bond to framework‐associated octahedral Al atoms. Replacement of an Al‐coordinated H_2_O molecule by 5‐FU is energetically favorable and the octahedral environment is further stabilized by the presence of additional, noncoordinated H_2_O molecules that form an extended hydrogen bond network. At least for the conditions and time scales probed in the simulations, no breaking of the Al−O(5‐FU) bond occurred, indicating that this coordination environment is stable at room temperature and in the presence of water. This is in stark contrast to prior observations made for 5‐FU adsorbed in protonic FAU containing Brønsted acid sites, where the bond(s) between 5‐FU and the framework proton(s) was (were) quickly broken if a cluster of H_2_O molecules was present in the vicinity.^[^ ^30^ ^]^ Interestingly, the qualitative difference in hydration stability is not correlated with the adsorption energies computed for water‐free models, which are more negative for 5‐FU interacting with framework protons. This discrepancy highlights the importance of considering competitive water adsorption when investigating different types of adsorption complexes in zeolites.

In the context of drug delivery applications, an essentially irreversible binding of 5‐FU to octahedral Al atoms is obviously undesirable as it limits the possible release of the drug under relatively mild conditions. In this regard, the results presented here support the conclusion by Datt et al., who attributed the limited release of 5‐FU from their zeolite samples to the formation of complexes between 5‐FU and octahedrally coordinated Al atoms.^[^ ^22^ ^]^ A comparison of DFT‐calculated IR spectra to experimental data showed much better agreement for those models in which 5‐FU is coordinated to Al_oct_ atoms, further corroborating the assumption that adsorption at these sites was an important, if not dominant process in the experimental samples. For the ^27^Al NMR spectra, the interpretation is not so straightforward, as the adsorption of 5‐FU mostly affects the line shape of the resonance that arises from octahedral Al atoms, whereas the isotropic chemical shift hardly changes. While the DFT‐based prediction of chemical shifts is straightforward, line shapes are influenced by a multitude of factors and therefore more challenging to compute. A general issue that affects the prediction of both IR and NMR spectra is the coexistence of many different local environments in real samples (different types of octahedral Al environments, different coordination modes of the guest molecule, etc.). As the calculations can typically cover only a few representative environments, the complexity of real samples cannot be captured in full and some deviations in calculated spectra are therefore not unexpected.^[^ ^63^ ^]^

Even though the calculations presented here considered only one framework type and a low Al content, it seems likely that the findings are largely transferable to other zeolite frameworks and Si/Al ratios due to the local nature of the Al−O(5‐FU) interaction. However, in structures with narrower pores and/or more Al atoms, it can be anticipated that Al‐coordinated 5‐FU molecules may be additionally stabilized, for example through bonds to adjacent Brønsted acid sites, hydrogen bonds to framework oxygen atoms, or possibly even simultaneous interaction with more than one octahedral Al atom. Similarly, it seems plausible to assume that the observations made for 5‐FU in the present study may also hold for other drug molecules that can bind to octahedral Al atom through carbonyl oxygen atoms (or, potentially, other functional groups with similar characteristics). When aiming to optimize the delivery of such molecules from zeolite hosts, it is crucial to minimize the amount of octahedral Al atoms, as an irreversible binding of the guest species to these sites will reduce the amount of drug that can be released.

From the viewpoint of an advanced microscopic understanding of zeolites, the present study illustrates the usefulness of recently derived models of framework‐associated octahedral Al sites in adsorption studies. While much of the computational work dealing with (extra‐framework or framework‐associated) Lewis acid sites in zeolites addresses their role in catalysis, much less emphasis is usually placed on their impact on adsorption properties. The case of 5‐FU adsorption in aluminosilicate FAU‐type zeolites demonstrates that the (essentially) irreversible binding of the guest molecule can only be reproduced when considering the presence of octahedral Al sites. It is anticipated that the explicit inclusion of such sites in computational studies will result in new atomic‐level insights into guest molecule adsorption in real, defect‐containing zeolites. In future work, the DFT‐based approach could be expanded towards transition state calculations or metadynamics simulations in order to obtain more direct insights into the mechanisms of exchange of Al‐coordinated guest molecules, such as the replacement of H_2_O by 5‐FU.

## Supporting Information

Supporting information PDF file contains details and parameters of force field calculations (**S1.1**, **S1.2**) and of DFT‐GIPAW (NMR) calculations (**S1.3**) as well as reporting further results of DFT optimizations (**S2.1** and **S2.2**), Mulliken population analysis (**S2.3**) AIMD simulations (**S2.4**), “composite” IR spectra (**S2.5**), and ^27^Al NMR shifts (**S2.6**). ZIP archives containing representative input files, optimized structures, results of vibrational calculations, and AIMD trajectories are available from Figshare: https://doi.org/10.6084/m9.figshare.28388117

## Conflict of Interests

The authors declare no conflict of interest.

## Supporting information



Supporting Information

## Data Availability

The data that support the findings of this study are openly available in Figshare at https://doi.org/10.6084/m9.figshare.28388117, reference number 28388117.

## References

[1] M. Vallet‐Regí , F. Balas , D. Arcos , Angew. Chemie ‐ Int. Ed. 2007, 46, 7548. 10.1002/anie.200604488 17854012

[2] M. Arruebo , Wiley Interdiscip. Rev. Nanomedicine Nanobiotechnology 2012, 4, 16. 21374827 10.1002/wnan.132

[3] E. Sayed , R. Haj‐Ahmad , K. Ruparelia , M. S. Arshad , M. W. Chang , Z. Ahmad , AAPS PharmSciTech 2017, 18, 1507. 28247293 10.1208/s12249-017-0740-2

[4] S. Mintova , M. Jaber , V. Valtchev , Chem. Soc. Rev. 2015, 44, 7207. 25983108 10.1039/c5cs00210a

[5] L. Bacakova , M. Vandrovcova , I. Kopova , I. Jirka , Biomater. Sci. 2018, 6, 974. 29630078 10.1039/c8bm00028j

[6] P. A. Wright , Microporous Framework Solids, Royal Society of Chemistry, Cambridge 2007.

[7] A. F. Masters , T. Maschmeyer , Microporous Mesoporous Mater. 2011, 142, 423.

[8] C. V. Uglea , I. Albu , A. Vatajanu , M. Croitoru , S. Antoniu , L. Panaitescu , R. M. Ottenbrite , J. Biomater. Sci. Polym. Ed. 1994, 6, 633. 7873514 10.1163/156856294x00572

[9] M. Jakubowski , M. Kucinska , M. Ratajczak , M. Pokora , M. Murias , A. Voelkel , M. Sandomierski , Microporous Mesoporous Mater. 2022, 343, 112194.

[10] M. Sandomierski , M. Jakubowski , M. Ratajczak , M. Pokora , M. Zielińska , A. Voelkel , J. Biomed. Mater. Res. ‐ Part B Appl. Biomater. 2023, 111, 1005. 10.1002/jbm.b.35209 36451589

[11] A. Domke , M. Fischer , M. Jakubowski , A. Pacholak , M. Ratajczak , A. Voelkel , M. Sandomierski , J. Drug Deliv. Sci. Technol. 2024, 99, 105997.

[12] P. Horcajada , C. Márquez‐Alvarez , A. Rámila , J. Pérez‐Pariente , M. Vallet‐Regí , Solid State Sci. 2006, 8, 1459.

[13] V. Mavrodinova , M. Popova , K. Yoncheva , J. Mihály , Á. Szegedi , J. Colloid Interface Sci. 2015, 458, 32. 26203589 10.1016/j.jcis.2015.07.026

[14] I. M. S. Souza , A. Borrego‐Sánchez , C. I. Sainz‐Díaz , C. Viseras , S. B. C. Pergher , Mater. Sci. Eng. C 2021, 118, 111365. 10.1016/j.msec.2020.111365 33254984

[15] A. Rossner , S. A. Snyder , D. R. U. Knappe , Water Res. 2009, 43, 3787. 19577267 10.1016/j.watres.2009.06.009

[16] D. J. De Ridder , J. Q. J. C. Verberk , S. G. J. Heijman , G. L. Amy , J. C. Van Dijk , Sep. Purif. Technol. 2012, 89, 71.

[17] S. Fukahori , T. Fujiwara , R. Ito , N. Funamizu , Desalination 2011, 275, 237.

[18] N. Jiang , R. Shang , S. G. J. Heijman , L. C. Rietveld , Water Res. 2018, 144, 145. 30025266 10.1016/j.watres.2018.07.017

[19] S. Vodenkova , T. Buchler , K. Cervena , V. Veskrnova , P. Vodicka , V. Vymetalkova , Pharmacol. Ther. 2020, 206, 107447. 31756363 10.1016/j.pharmthera.2019.107447

[20] J. L. Arias , Molecules 2008, 13, 2340. 18830159 10.3390/molecules13102340 PMC6245407

[21] A. A. Valencia‐Lazcano , D. Hassan , M. Pourmadadi , A. Shamsabadipour , R. Behzadmehr , A. Rahdar , D. I. Medina , A. M. Díez‐Pascual , Eur. J. Med. Chem. 2023, 246, 114995. 36493619 10.1016/j.ejmech.2022.114995

[22] A. Datt , E. A. Burns , N. A. Dhuna , S. C. Larsen , Microporous Mesoporous Mater. 2013, 167, 182.

[23] M. Ravi , V. L. Sushkevich , J. A. Van Bokhoven , J. Phys. Chem. C 2019, 123, 15139.

[24] M. Ravi , V. L. Sushkevich , J. A. van Bokhoven , Nat. Mater. 2020, 19, 1047. 32958864 10.1038/s41563-020-0751-3

[25] N. Vilaça , R. Amorim , A. F. Machado , P. Parpot , M. F. R. Pereira , M. Sardo , J. Rocha , A. M. Fonseca , I. C. Neves , F. Baltazar , Colloids Surf., B 2013, 112, 237. 10.1016/j.colsurfb.2013.07.042 23988779

[26] M. Spanakis , N. Bouropoulos , D. Theodoropoulos , L. Sygellou , S. Ewart , A. M. Moschovi , A. Siokou , I. Niopas , K. Kachrimanis , V. Nikolakis , P. A. Cox , I. S. Vizirianakis , D. G. Fatouros , Nanomed. Nanotechnol., Biol. Med. 2014, 10, 197. 10.1016/j.nano.2013.06.016 23916887

[27] N. Vilaça , A. F. Machado , F. Morais‐Santos , R. Amorim , A. Patrícia Neto , E. Logodin , M. F. R. Pereira , M. Sardo , J. Rocha , P. Parpot , A. M. Fonseca , F. Baltazar , I. C. Neves , RSC Adv. 2017, 7, 13104.

[28] O. Y. Golubeva , Y. A. Alikina , E. Y. Brazovskaya , V. V. Ugolkov , Appl. Clay Sci. 2020, 184, 105401.

[29] A. R. Bertão , V. Ivasiv , C. Almeida‐Aguiar , P. R. Correia , A. M. Fonseca , M. Bañobre‐López , F. Baltazar , I. C. Neves , Microporous Mesoporous Mater. 2024, 364, 112871.

[30] M. Fischer , CrystEngComm 2024, 26, 3795.

[31] S. Li , A. Zheng , Y. Su , H. Fang , W. Shen , Z. Yu , L. Chen , F. Deng , Phys. Chem. Chem. Phys. 2010, 12, 3895. 20358084 10.1039/b915401a

[32] M. C. Silaghi , C. Chizallet , P. Raybaud , Microporous Mesoporous Mater. 2014, 191, 82.

[33] M. C. Silaghi , C. Chizallet , J. Sauer , P. Raybaud , J. Catal. 2016, 339, 242.

[34] E. V. Khramenkova , H. Venkatraman , V. Soethout , E. A. Pidko , Phys. Chem. Chem. Phys. 2022, 138, 27047. 10.1039/d2cp03603g PMC9673684 36321744

[35] M. Jin , M. Ravi , C. Lei , C. J. Heard , F. Brivio , Z. Tošner , L. Grajciar , J. A. van Bokhoven , P. Nachtigall , Angew. Chemie Int. Ed. 2023, 62, e202306183. 10.1002/anie.202306183 37283089

[36] J. L. Mancuso , V. Van Speybroeck , J. Catal. 2024, 429, 115211.

[37] M. J. Sanders , M. Leslie , C. R. A. Catlow , J. Chem. Soc. Chem. Commun. 1984, 1271.

[38] J. D. Gale , A. L. Rohl , Mol. Simul. 2003, 29, 291.

[39] M. Waldman , A. T. Hagler , J. Comput. Chem. 1993, 14, 1077.

[40] H. Sun , S. J. Mumby , J. R. Maple , A. T. Hagler , J. Am. Chem. Soc. 1994, 116, 2978.

[41] F. S. Emami , V. Puddu , R. J. Berry , V. Varshney , S. V. Patwardhan , C. C. Perry , H. Heinz , Chem. Mater. 2014, 26, 2647.

[42] S. Kim , J. Chen , T. Cheng , A. Gindulyte , J. He , S. He , Q. Li , B. A. Shoemaker , P. A. Thiessen , B. Yu , L. Zaslavsky , J. Zhang , E. E. Bolton , Nucleic Acids Res. 2023, 51, D1373. 36305812 10.1093/nar/gkac956 PMC9825602

[43] J. Wielińska , A. Nowacki , B. Liberek , Molecules 2019, 24, 3683. 31614932 10.3390/molecules24203683 PMC6832121

[44] J. VandeVondele , M. Krack , F. Mohamed , M. Parrinello , T. Chassaing , J. Hutter , Comput. Phys. Commun. 2005, 167, 103. 10.1063/1.1828433 15638682

[45] T. D. Kühne , M. Iannuzzi , M. Del Ben , V. V. Rybkin , P. Seewald , F. Stein , T. Laino , R. Z. Khaliullin , O. Schütt , F. Schiffmann , D. Golze , J. Wilhelm , S. Chulkov , M. H. Bani‐Hashemian , V. Weber , U. Borštnik , M. Taillefumier , A. S. Jakobovits , A. Lazzaro , H. Pabst , T. Müller , R. Schade , M. Guidon , S. Andermatt , N. Holmberg , G. K. Schenter , A. Hehn , A. Bussy , F. Belleflamme , G. Tabacchi , et al., J. Chem. Phys. 2020, 152, 194103. 33687235 10.1063/5.0007045

[46] J. VandeVondele , J. Hutter , J. Chem. Phys. 2007, 127, 114105. 17887826 10.1063/1.2770708

[47] M. Krack , Theor. Chem. Acc. 2005, 114, 145.

[48] I. Hamada , Phys. Rev. B 2014, 89, 121103.

[49] M. Fischer , J. Brauer , ChemistryOpen 2024, 7, e202300273. 10.1002/open.202300273 PMC11230941 38385822

[50] K. Momma , F. Izumi , J. Appl. Crystallogr. 2011, 44, 1272.

[51] G. Schaftenaar , J. H. Noordik , J. Comput. Aided. Mol. Des. 2000, 14, 123. 10721501 10.1023/a:1008193805436

[52] G. Schaftenaar , E. Vlieg , G. Vriend , J. Comput. Aided. Mol. Des. 2017, 31, 789. 28752344 10.1007/s10822-017-0042-5 PMC5633641

[53] S. Nosé , J. Chem. Phys. 1984, 81, 511.

[54] W. G. Hoover , Phys. Rev. A 1985, 31, 1695. 10.1103/physreva.31.1695 9895674

[55] S. J. Clark , M. D. Segall , C. J. Pickard , P. J. Hasnip , M. I. J. Probert , K. Refson , M. C. Payne , Zeitschrift für Krist 2005, 220, 567.

[56] V. Milman , K. Refson , S. J. Clark , C. J. Pickard , J. R. Yates , S.‐P. Gao , P. J. Hasnip , M. I. J. Probert , A. Perlov , M. D. Segall , J. Mol. Struct. Theochem. 2010, 954, 22.

[57] M. D. Segall , R. Shah , C. J. Pickard , M. C. Payne , Phys. Rev. B 1996, 54, 16317. 10.1103/physrevb.54.16317 9985733

[58] C. J. Pickard , F. Mauri , Phys. Rev. B 2001, 63, 245101.

[59] J. R. Yates , C. J. Pickard , F. Mauri , Phys. Rev. B 2007, 76, 024401.

[60] J. P. Perdew , K. Burke , M. Ernzerhof , Phys. Rev. Lett. 1996, 77, 3865. 10062328 10.1103/PhysRevLett.77.3865

[61] D. Willimetz , A. Erlebach , C. J. Heard , L. Grajciar , Digit. Discov. 2025, 4, 275.

[62] S. Alvarez , Dalton Trans. 2013, 42, 8617. 23632803 10.1039/c3dt50599e

[63] M. Fischer , R. Fantini , R. Arletti , J. Brauer , L. Mino , J. Phys. Chem. C 2023, 117, 24242.

