# Supplementary material for: Impact of Octahedral Aluminum Sites on Guest Molecule Adsorption in Zeolites: A Computational Study of 5‐Fluorouracil in Zeolite FAU
Source: Chemistry. 2025 May 2;31(32):e202500833. doi: 10.1002/chem.202500833 (PMC12144882; doi:10.1002/chem.202500833)
Supplement: Supplementary file 1 — Supporting Information [file CHEM-31-e202500833-s001.pdf]

Impact of Octahedral Aluminum Sites on Guest Molecule Adsorption in Zeolites: A Computational Study of 5-Fluorouracil in Zeolite FAU

Michael Fischer

Faculty of Geosciences, University of Bremen, Klagenfurter Straße 2-4, 28359 Bremen, Germany

Bremen Center for Computational Materials Science (BCCMS) and MAPEX Center for Materials and Processes, University of Bremen, 28359 Bremen, Germany

E-mail: michael.fischer@uni-bremen.de

SUPPORTING INFORMATION

Contents

S1.1 Details of simulated annealing simulations **S2**

S1.2 Force field parameters **S2**

S1.3 Details of DFT-GIPAW calculations of ^{27}Al NMR chemical shifts **S4**

S2.1 Results of DFT optimizations **S5**

S2.2 Results of DFT optimizations including cell parameter optimization **S7**

S2.3 Mulliken population analysis **S8**

S2.4 Results of AIMD simulations **S11**

S2.5 DFT-calculated “composite” IR spectrum **S15**

S2.6 Results of DFT-GIPAW calculations **S16**

S1.1 Details of simulated annealing simulations

To study models with co-adsorbed water molecules, simulated annealing simulations were employed to add H_2O molecules to previously optimized models of FAU_fwAl$_{oct}$_2Al. In the context of the calculations reported in **3.1**, 6 H_2O molecules were added to the initial FAU_fwAl$_{oct}$_2Al model (without 5-FU). For the calculations described in **3.4**, 14 H_2O molecules were added to the two low-energy 5-FU@FAU_fwAl$_{oct}$_2Al configurations (1_O4 and 2_O2). The simulations used the *Adsorption Locator* module of the DS BIOVIA *Materials Studio* suite, which employs a simulated annealing procedure that is based on a series of Monte Carlo simulations during which the temperature is gradually decreased from a very high starting value (100,000 K) to low temperature (100 K), thus allowing to probe different local minima. After an initial loading of the specified number of H_2O molecules into the simulation cell, each simulated annealing run consisted of 10 cycles, with at least 200,000 simulation steps per cycle, using the following probability ratios for different types of MC moves: Translation:Rotation:Regrowth = 1:1:0.1. The structures of the host system (FAU_fwAl$_{oct}$_2Al ± 5-FU) were held fixed during the simulations, and the geometry of the H_2O molecule, previously optimized with PCFF force field parameters,[1] was also held fixed.

The Lennard-Jones (LJ) parameters and partial charges used to model host-guest and guest-guest interactions are summarized in the following section. The Ewald & Group method was used for Coulomb interactions, using an accuracy of 0.0001 kcal mol^{-1} and a cutoff distance of 15.5 Å, whereas an atom-based summation was used for the Lennard-Jones potentials, employing a cutoff of 15.5 Å and a spline-based truncation with a spline width of 1 Å.

To ascertain that the additional H_2O molecules are adsorbed in the vicinity of the octahedral Al site, an additional constraint was imposed in the form of an “adsorption distance” (maximal distance between added H_2O molecules and Al-coordinated H_2O/5-FU molecules), which was set to 6 Å. Five independent simulated annealing runs were performed for each case of interest (FAU_fwAl$_{oct}$_2Al, 5-FU@FAU_fwAl$_{oct}$_2Al [5-FU_1_O4], 5-FU@FAU_fwAl$_{oct}$_2Al [5-FU_2_O2]). At the end of each annealing cycle, the positions of the H_2O molecules were optimized, using the *Forcite* module of *Materials Studio* and employing “fine” optimization settings (energy convergence: 10^{-4} kcal mol^{-1}, force convergence: 0.005 kcal mol^{-1} Å^{-1}, maximal displacement between steps: $5 \cdot 10^{-5}$ Å). Out of the 50 configurations generated for $(H_2O)_6$@FAU_fwAl$_{oct}$_2Al, five were selected and DFT-optimized, and the lowest-energy configuration was taken as starting point for the AIMD simulations reported in **3.1**. For the $(H_2O)_{14}$@(5-FU@FAU_fwAl$_{oct}$_2Al) systems considered in **3.4**, six out of the 100 configurations were selected and taken as starting points for AIMD simulations (three for 5-FU_1_O4, three for 5-FU_2_O2).

S1.2 Force field parameters

5-FU and H_2O molecules were represented using default parameters from the PCFF force field that are distributed with *Materials Studio* (**Table S1**).[1] PCFF uses 9-6 LJ potentials and a 6th-power combination rule.[2] Regarding the zeolite framework, partial charges and LJ parameters proposed by Emami et al. were employed for Si atoms and O atoms of Si−O−Si linkages,[3] while parameters developed in the previous study of 5-FU adsorption in protonated zeolites were used for tetrahedral Al atoms, oxygen atoms of Si−O−Al linkages, and protons bonded to O atoms of Si−O−Al linkages.[4] LJ parameters and charges of the O and H atom of the silanol group were, again, taken from the PCFF force field. For the remaining two atom types,

the octahedral Al atom and the Si atom neighboring the silanol group, the same LJ parameters as for other Al/Si atoms were used, but the charges were slightly adjusted to give overall charge neutrality.

Table S1: Atom types and non-bonded PCFF parameters used in force field calculations. Partial charges q and Lennard-Jones parameters R_0 and D_0 are given for each atom. NI (no interaction) indicates that only a partial charge, but no LJ potentials were assigned to this atom.

**5-FU molecule**	**Atom type (PCFF)**	q **[*e*]**	R_0 **[Å]**	D_0 **[kcal mol^{-1}]**
C2	c_2	0.807	3.81	0.120
C4	c_1	0.642	3.81	0.120
C5	c=1	0.130	4.01	0.064
C6	c=	-0.0268	3.90	0.064
N1	n_2	-0.589	3.60	0.106
N3	n_2	-0.600	3.60	0.106
O2	o_1	-0.585	3.30	0.267
O4	o_1	-0.531	3.30	0.267
F	f	-0.130	3.20	0.0598
H1, H3	hn2	0.378	1.65	0.013
H6	hc	0.1268	NI	NI
**H_2O molecule**				
O	o*	-0.7982	3.608	0.274
H	hw	0.3991	1.098	0.013
**Zeolite**				
Si_{tet}	sz	1.100	4.20	0.080
$Si_{silanol}$	sz	1.2303	4.20	0.080
Al_{tet}	az	1.130	4.20	0.080
Al_{oct}	az	1.060	4.20	0.080
$O_{Si-O-Si}$	oss	-0.550	3.60	0.040
$O_{Si-O-Al}$ (not protonated)	oas	-0.720	3.60	0.040
$O_{Si-O-Al}$ (protonated)	ob	-0.510	3.60	0.040
$O_{silanol}$	osh	-0.1944	3.4618	0.1591
$H_{Si-O-Al}$	hb	0.440	1.098	0.013
$H_{silanol}$	hos	0.0641	2.3541	0.0988

S1.3 Details of DFT-GIPAW calculations of ^{27}Al NMR chemical shifts

DFT calculations to predict ^{27}Al NMR chemical shifts used the gauge-including projector augmented-wave (GIPAW) method[5,6] as implemented in the CASTEP code.[7,8] All NMR calculations used the structures previously optimized with CP2K (rev-vdW-DF2 functional, TZVP basis sets) without additional optimization with CASTEP. The PBE exchange-correlation functional was used.[9] The plane-wave cutoff energy was set to 600 eV, and on-the-fly-generated ultrasoft pseudopotentials were used. In the view of the size of the FAU unit cell, the first Brillouin zone was sampled at the Γ point, only.

The DFT-GIPAW calculations deliver isotropic chemical shieldings σ_{iso}. To convert the ^{27}Al chemical shieldings into chemical shifts δ_{iso}, the equation proposed in a recent computational study of ^{27}Al NMR chemical shifts in zeolites by Willimetz et al. was used:[10]

$$\delta_{\mathrm{iso}}(\mathrm{Al}) = -\sigma_{\mathrm{iso}}(\mathrm{Al}) + 549.8\ \mathrm{ppm}$$

Willimetz et al. employed very similar computational settings to compute the isotropic chemical shieldings as the present work. The transferability of their equation is corroborated by the good agreement of the calculated chemical shifts with the experimental values observed by Datt et al. for both tetrahedral and octahedral Al atoms.[11]

S2.1 Results of DFT optimizations

Table S2: Results of DFT optimizations: DFT total energies and, where applicable, energy differences ΔE_{DFT} and/or adsorption energies ΔE_{ads}.

Unit conversion: 1 Hartree (Ha) = 2625.4996 kJ mol^{-1}.

Table S2 a) Guest molecules

**Molecule**	$\mathbf{E_{DFT}}$ **[Ha]**
5-FU	-100.80831
H_2O	-17.23700

Table S2 b) FAU_fwAl$_{oct}$_2Al (three Al-coordinated H_2O molecules)

**Model**	$\mathbf{E_{DFT}}$ **[Ha]**	$\mathbf{\Delta E_{DFT}}$ **[kJ mol^{-1}]**
Initial structure	−6999.83203	0
Lowest-energy*	−6999.83214	−0.3

*Optimized snapshot 17,500 from AIMD trajectory for T = 298 K

Table S2 c) FAU_fwAl$_{tbp}$_2Al (two Al-coordinated H_2O molecules)

**Model**	$\mathbf{E_{DFT}}$ **[Ha]**	$\mathbf{\Delta E_{DFT}}$ **[kJ mol^{-1}]**
Removal of H_2O(1)	−6982.569411	0.1
Removal of H_2O(2)	−6982.560169	24.4
Removal of H_2O(3)	−6982.569451	0

Table S2 d) FAU_fwAl$_{oct}$_2Al with six additional (non-coordinated) H_2O molecules

**Model**	$\mathbf{E_{DFT}}$ **[Ha]**	$\mathbf{\Delta E_{DFT}}$ **[kJ mol^{-1}]**	$\mathbf{E_{ads}}$ **[kJ mol^{-1}]***
Config1 from GCMC	−7103.39699	10.0	−62.5
Config2 from GCMC	−7103.40081	0	−64.2
Config3 from GCMC	−7103.39424	17.3	−61.3
Config4 from GCMC	−7103.39703	9.9	−62.5
Config5 from GCMC	−7103.38772	34.4	−58.4

*Calculated per added H_2O molecule, using lowest-energy FAU_fwAl$_{oct}$_2Al model as reference

Table S2 e) 5-FU@FAU_fwAl$_{oct}$_2Al

Index	Replaced molecule	5-FU coordination	E_{DFT} [Ha]	ΔE_{DFT} [kJ mol^{-1}]	E_{ads} [kJ mol^{-1}]*
1_O2	H_2O(1)	O2-on	−7083.41837	12.1	−106.6
1_O4	H_2O(1)	O4-on	−7083.42296	0	−118.7
2_O2	H_2O(2)	O2-on	−7083.42241	1.4	−117.2
2_O4	H_2O(2)	O4-on	−7083.42129	4.4	−114.3
3_O2	H_2O(3)	O2-on	−7083.42065	6.1	−112.6
3_O4	H_2O(3)	O4-on	−7083.42140	4.1	−114.6

*Calculated according to equation (1) of the main paper

Table S2 f) FAU with Brønsted acid site (H-FAU_1H)

Model	E_{DFT} [Ha]	E_{ads} [kJ mol^{-1}]
H-FAU_1H	−6949.32271	./.
5-FU@H-FAU_1H*	−7050.18661	−146.0

* Six initial adsorption configurations were DFT-optimized, only the results for the lowest-energy structure are included.

S2.2 Results of DFT optimizations including cell parameter optimization

The calculations reported in the main paper always fixed the cell parameters to the optimized parameters of all-silica FAU (a = 24.227 Å), which were obtained from GULP calculations using force field parameters proposed by Sanders, Leslie, and Catlow (SLC).[12,13] In order to verify that variations in the equilibrium cell parameters among the different systems are not significant, structure optimizations including a relaxation of the unit cell parameters were carried out for selected structures, fixing the unit cell to a cubic metric. The results of these calculations are summarized in **Table S3**. While it is evident that the cell parameters increase by about 0.2 Å (~0.8%) compared to the SLC-optimized parameters, the equilibrium cell parameters are hardly affected by the guest molecule content: All DFT-optimized values of a fall in an interval from 24.418 Å to 24.435 Å (Δa = 0.017 Å) regardless of the absence/presence of 5-FU and/or non-coordinated water molecules. This indicates that any cell distortion due to the presence of guest molecules remains negligible at the low loadings considered here. While the average adsorption energy for non-coordinated H_2O molecule is practically indistinguishable from the corresponding value given in **Table S2 d**, computed 5-FU adsorption energies show a more significant deviation from the results obtained with the fixed cell. However, this observation should not be overinterpreted as only two 5-FU positions and coordination modes were included in the calculations that relaxed the cell parameters. Altogether, it is expected that the main qualitative findings are not affected by the use of fixed, previously optimized cell parameters.

Table S3: DFT-optimized unit cell parameters and, where applicable, adsorption energies of selected models.

**Model**	E_{DFT} **[Ha]**	a **[Å]**	E_{ads} **[kJ mol^{-1}]**
FAU_fwAl$_{oct}$_2Al	−6999.87165	24.428	./.
FAU_fwAl$_{oct}$_2Al + 6 H_2O [Config 2]	−7103.44158	24.431	−64.7
FAU_fwAl$_{tbp}$_2Al [H_2O(3) removed]	−6982.61034	24.430	./.
5-FU@FAU_fwAl$_{oct}$_2Al (5-FU_1_O4)	−7083.46527	24.435	−122.4
5-FU@FAU_fwAl$_{oct}$_2Al (5-FU_2_O2)	−7083.45999	24.422	−108.5
5-FU@FAU_fwAl$_{oct}$_2Al (5-FU_1_O4) + 14 H_2O			
Config1 from GCMC	−7325.07625	24.422	./.
Config2 from GCMC	−7325.09086	24.428	./.
Config3 from GCMC	−7325.07798	24.425	./.
5-FU@FAU_fwAl$_{oct}$_2Al (5-FU_2_O2) + 14 H_2O			
Config1 from GCMC	−7325.10149	24.426	./.
Config2 from GCMC	−7325.09150	24.418	./.
Config3 from GCMC	−7325.103226	24.421	./.

S2.3 Mulliken population analysis

For selected FAU models, a Mulliken population analysis was carried out using CASTEP. **Table S4 a** includes the population of the 3*s* and 3*p* orbitals of the Al atoms and the resulting Mulliken charges. **Table S4 b** to **S4 g** summarize the results of the overlap (bond) population analysis for all Al−O bonds in the respective models.

Table S4 a: Population of 3*s* and 3*p* orbitals of Al atoms and Mulliken charges (in *e*).

**Model**	**Al_{tet}**			**Al_{oct}**		
	**3*s***	**3*p***	**q_{Mull}**	**3*s***	**3*p***	**q_{Mull}**
FAU_fwAl$_{oct}$_1Al				0.422	0.606	1.971
FAU_fwAl$_{oct}$_2Al*	0.385	0.585	2.031	0.425	0.579	1.996
FAU_fwAl$_{oct}$_2Al + 6 H_2O*	0.383	0.584	2.033	0.428	0.601	1.970
5-FU@FAU_fwAl$_{oct}$_2Al (5-FU_1_O4)	0.383	0.582	2.034	0.414	0.581	2.005
5-FU@FAU_fwAl$_{oct}$_2Al (5-FU_2_O2)	0.385	0.584	2.032	0.422	0.586	1.993
FAU_fwAl$_{tbp}$_2Al	0.383	0.567	2.049	0.390	0.541	2.069

* Lowest-energy structure obtained from DFT optimization of AIMD snapshot

Table S4 b: Overlap population analysis of FAU_fwAl$_{oct}$_1Al.

**Al_{oct}**	***d* [Å]**	**Overlap population [|e|]**
Al_{oct}−O_{fw}	1.779	0.40
Al_{oct}−O_{fw}	1.780	0.39
Al_{oct}−O_{fw}	1.785	0.36
Al_{oct}−O(H_2O(1))	2.068	0.14
Al_{oct}−O(H_2O(2))	2.070	0.16
Al_{oct}−O(H_2O(3))	2.081	0.17

Table S4 c: Overlap population analysis of FAU_fwAl$_{oct}$_2Al.

**Al$_{tet}$**	***d* [Å]**	**Overlap population [|*e*|]**	**Al$_{oct}$**	***d* [Å]**	**Overlap population [|*e*|]**
Al$_{tet}$–O$_{fw}$	1.723	0.41	Al$_{oct}$–O$_{fw}$	1.769	0.39
Al$_{tet}$–O$_{fw}$	1.732	0.40	Al$_{oct}$–O$_{fw}$	1.786	0.40
Al$_{tet}$–O$_{fw}$	1.746	0.41	Al$_{oct}$–O$_{fw}$(–H)	1.939	0.22
Al$_{tet}$–O$_{fw}$(···H)	1.792	0.32	Al$_{oct}$–O(H_2O(1))	1.992	0.18
			Al$_{oct}$–O(H_2O(2))	2.000	0.17
			Al$_{oct}$–O(H_2O(3))	2.043	0.21

Table S4 d: Overlap population analysis of FAU_fwAl$_{oct}$_2Al + 6 H_2O.

**Al$_{tet}$**	***d* [Å]**	**Overlap population [|*e*|]**	**Al$_{oct}$**	***d* [Å]**	**Overlap population [|*e*|]**
Al$_{tet}$–O$_{fw}$	1.725	0.35	Al$_{oct}$–O$_{fw}$	1.761	0.38
Al$_{tet}$–O$_{fw}$	1.726	0.43	Al$_{oct}$–O$_{fw}$	1.774	0.37
Al$_{tet}$–O$_{fw}$	1.734	0.38	Al$_{oct}$–O$_{fw}$(–H)	2.029	0.24
Al$_{tet}$–O$_{fw}$(···H)	1.803	0.39	Al$_{oct}$–O(H_2O(1))	2.018	0.19
			Al$_{oct}$–O(H_2O(2))	2.020	0.22
			Al$_{oct}$–O(H_2O(3))	1.945	0.21

Table S4 e: Overlap population analysis of 5-FU@FAU_fwAl$_{oct}$_2Al (5-FU_1_O4).

**Al$_{tet}$**	***d* [Å]**	**Overlap population [|*e*|]**	**Al$_{oct}$**	***d* [Å]**	**Overlap population [|*e*|]**
Al$_{tet}$–O$_{fw}$	1.720	0.42	Al$_{oct}$–O$_{fw}$	1.761	0.37
Al$_{tet}$–O$_{fw}$	1.739	0.41	Al$_{oct}$–O$_{fw}$	1.807	0.39
Al$_{tet}$–O$_{fw}$	1.741	0.41	Al$_{oct}$–O$_{fw}$(–H)	1.944	0.20
Al$_{tet}$–O$_{fw}$(···H)	1.782	0.33	Al$_{oct}$–O4(5-FU)	1.914	0.27
			Al$_{oct}$–O(H_2O(2))	2.023	0.17
			Al$_{oct}$–O(H_2O(3))	2.034	0.21

Table S4 f: Overlap population analysis of 5-FU@FAU_fwAl_{oct}_2Al (5-FU_2_O2).

**Al_{tet}**	***d* [Å]**	**Overlap population [|*e*|]**	**Al_{oct}**	***d* [Å]**	**Overlap population [|*e*|]**
Al_{tet}–O_{fw}	1.723	0.41	Al_{oct}–O_{fw}	1.785	0.38
Al_{tet}–O_{fw}	1.736	0.4	Al_{oct}–O_{fw}	1.800	0.39
Al_{tet}–O_{fw}	1.745	0.41	Al_{oct}–O_{fw}(–H)	1.962	0.21
Al_{tet}–O_{fw}(···H)	1.794	0.32	Al_{oct}–O2(5-FU)	1.919	0.26
			Al_{oct}–O(H_2O(1))	2.007	0.18
			Al_{oct}–O(H_2O(3))	2.044	0.21

Table S4 g: Overlap population analysis of FAU_fwAl_{tbp}_2Al.

**Al_{tet}**	***d* [Å]**	**Overlap population [|*e*|]**	**Al_{oct}**	***d* [Å]**	**Overlap population [|*e*|]**
Al_{tet}–O_{fw}	1.722	0.41	Al_{tbp}–O_{fw}	1.718	0.42
Al_{tet}–O_{fw}	1.731	0.41	Al_{tbp}–O_{fw}	1.725	0.44
Al_{tet}–O_{fw}	1.741	0.41	Al_{tbp}–O_{fw}(–H)	1.916	0.21
Al_{tet}–O_{fw}(···H)	1.804	0.29	Al_{tbp}–O(H_2O(1))	1.857	0.25
			Al_{tbp}–O(H_2O(2))	2.037	0.17

S2.4 Results of AIMD simulations

Optimization of snapshots from AIMD simulations: Snapshots were extracted from the AIMD trajectories every 2,500 steps (= every 1.25 ps) and fully optimized using DFT. **Figures S1** to **S3** visualize the energy differences ΔE_{DFT} for series of snapshots.

Figure S1: AIMD simulations of FAU_fwAl$_{oct}$_2Al

The reference energy is taken from the initial optimized structure, E_{DFT} = −6999.83203 Ha (**Table S2 b**).

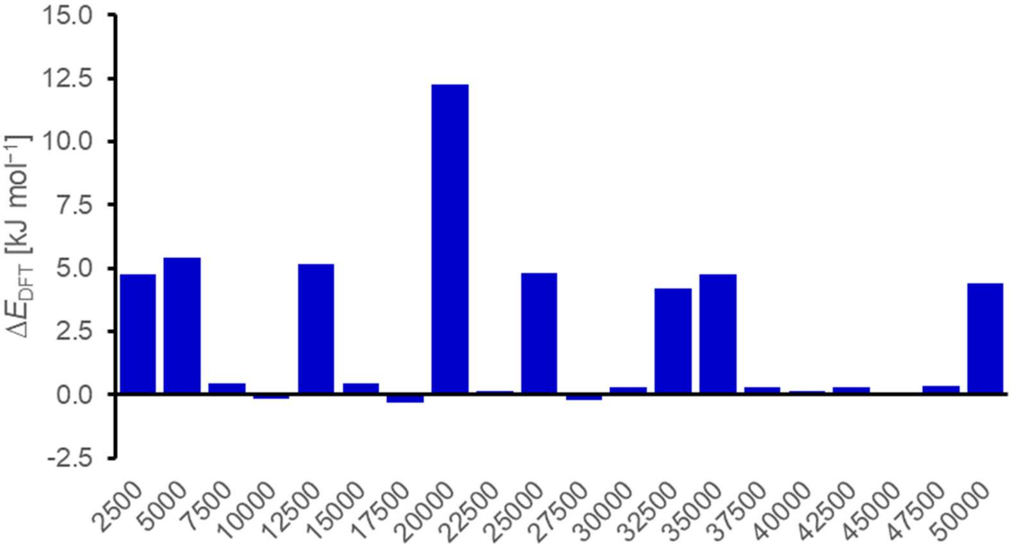

Figure S1a: ΔE_{DFT} of optimized snapshots of FAU_fwAl$_{oct}$_2Al obtained for T = 298 K

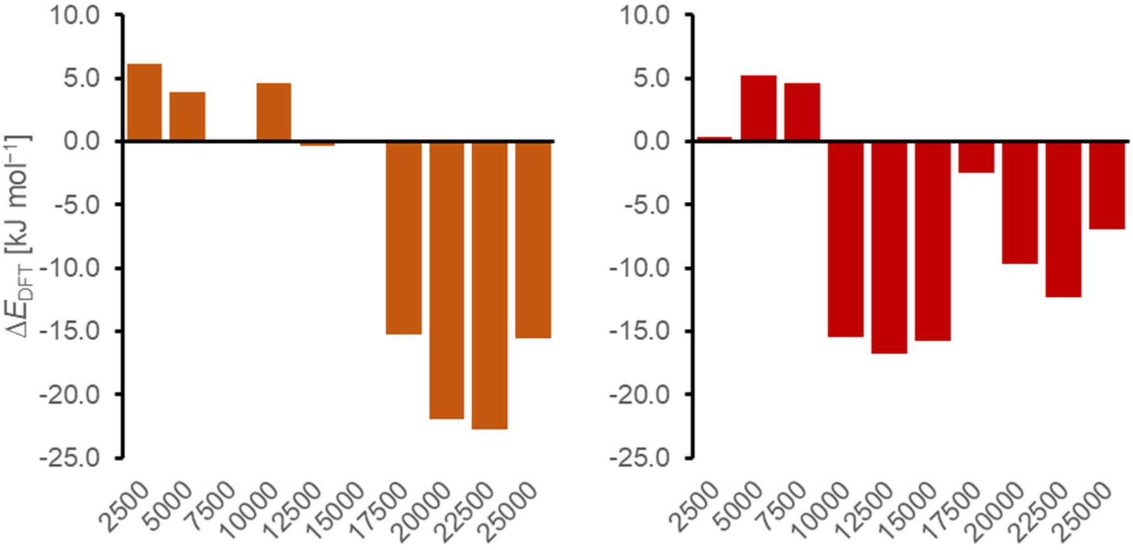

Figure S1b: ΔE_{DFT} of optimized snapshots of FAU_fwAl$_{oct}$_2Al obtained for T = 373 K (left) and 473 K (right). Dissociation of one Al−O(H_2O) bond takes place after about 15,000 steps (373 K) and 7,500 steps (473 K), respectively (**Figure 2** of main paper).

Figure S2: AIMD simulations of FAU_fwAl$_{oct}$_2Al + 6 H_2O

The reference energy is taken from the lowest-energy DFT-optimized structure generated with GCMC simulations, E_{DFT} = −7103.40081 Ha (Config2 in **Table S2 d**).

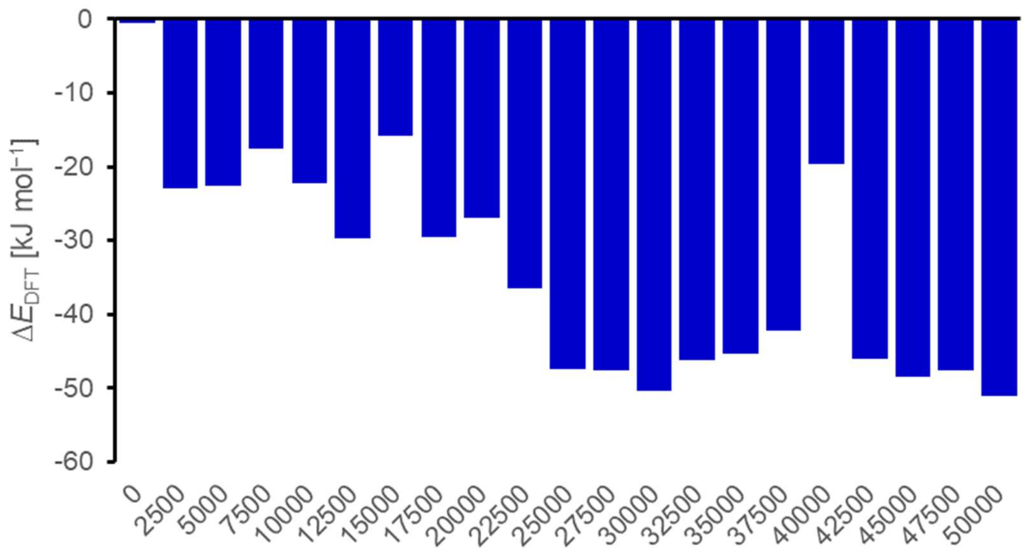

Figure S2a: ΔE_{DFT} of optimized snapshots of FAU_fwAl$_{oct}$_2Al + 6 H_2O obtained for T = 298 K. **Figure 3** of the main paper shows the optimized snapshot at step 50,000.

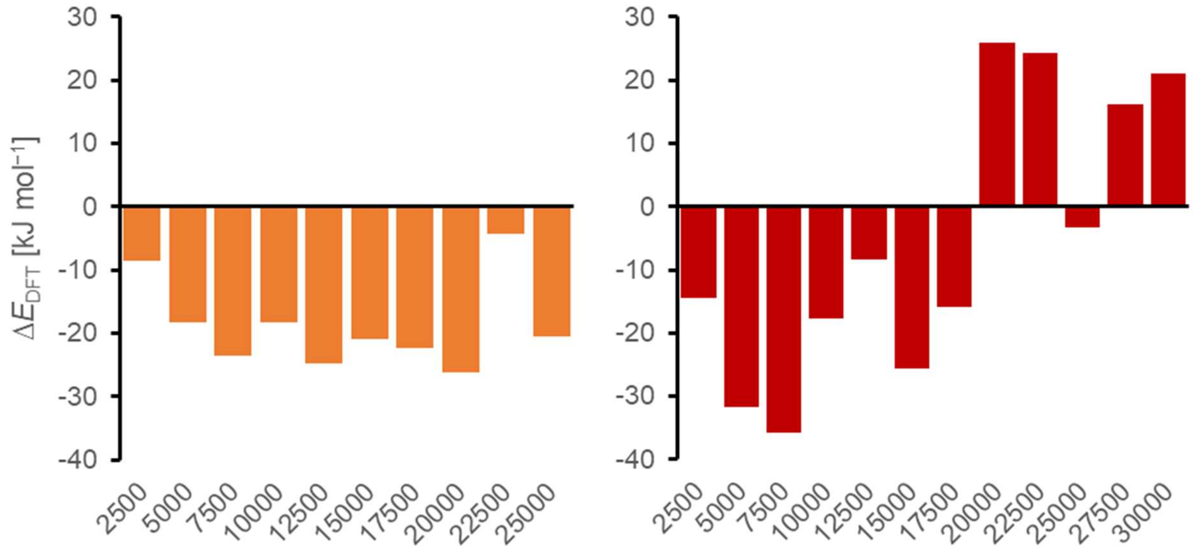

Figure S2b: ΔE_{DFT} of optimized snapshots of FAU_fwAl$_{oct}$_2Al + 6 H_2O obtained for T = 373 K (left) and 473 K (right).

Figure S3: AIMD simulations of 5-FU@FAU_fwAl$_{oct}$_2Al

The reference energy is taken from the lowest-energy DFT-optimized structure of 5-FU@FAU_fwAl$_{oct}$_2Al, index 1_O4 in **Table S2 e**, E_{DFT} = −7083.42296 Ha.

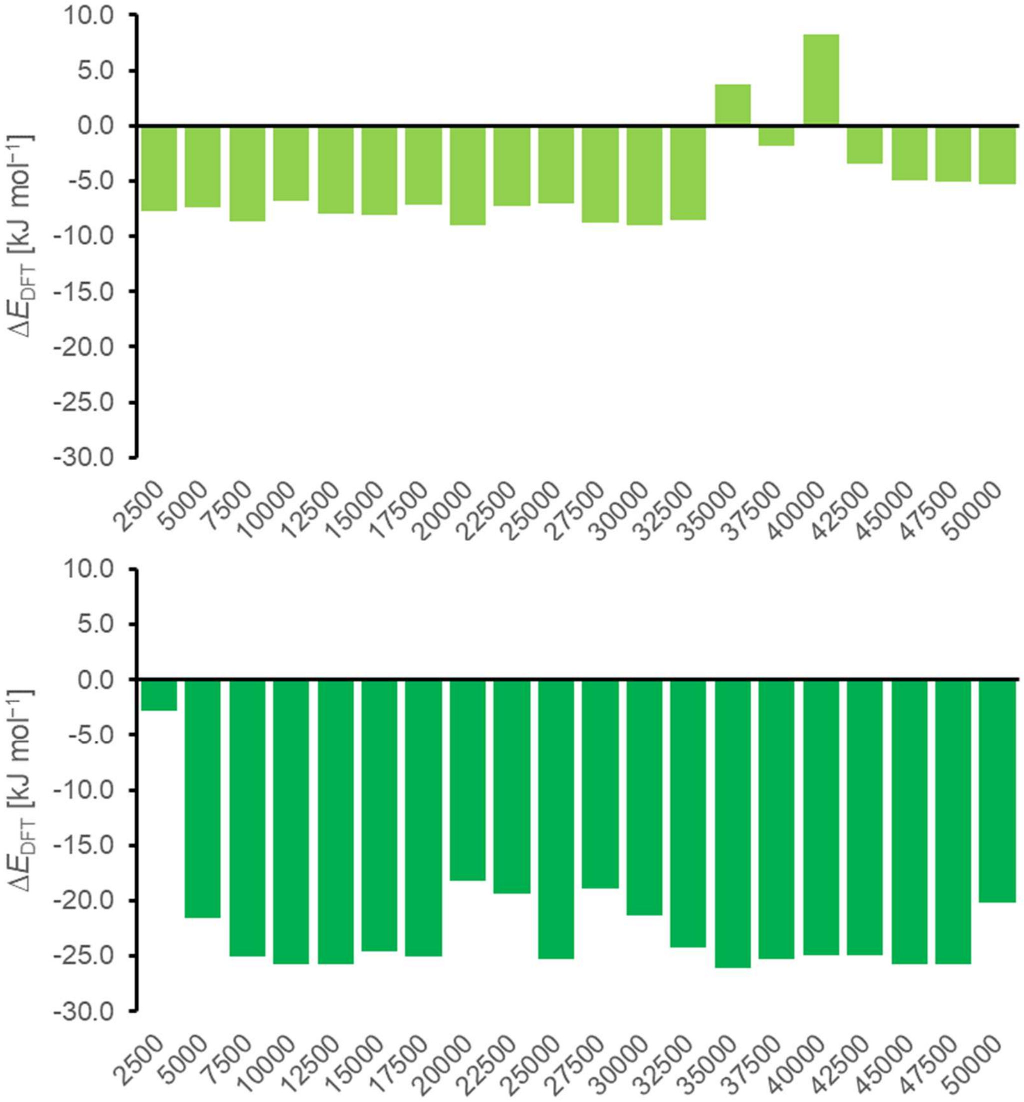

Figure S3: ΔE_{DFT} of optimized snapshots of 5-FU@FAU_fwAl$_{oct}$_2Al obtained for T = 298 K. Top: configuration with index 1_O4 (dissociation of one Al−O(H_2O) bond after about 35,000 steps, see **Figure 6a**), bottom: configuration with index 2_O2 (dissociation of one Al−O(H_2O) bond after about 5,000 steps, see **Figure 6b**). **Figure 6c** shows the optimized snapshot of configuration O2_2 after 35,000 steps.

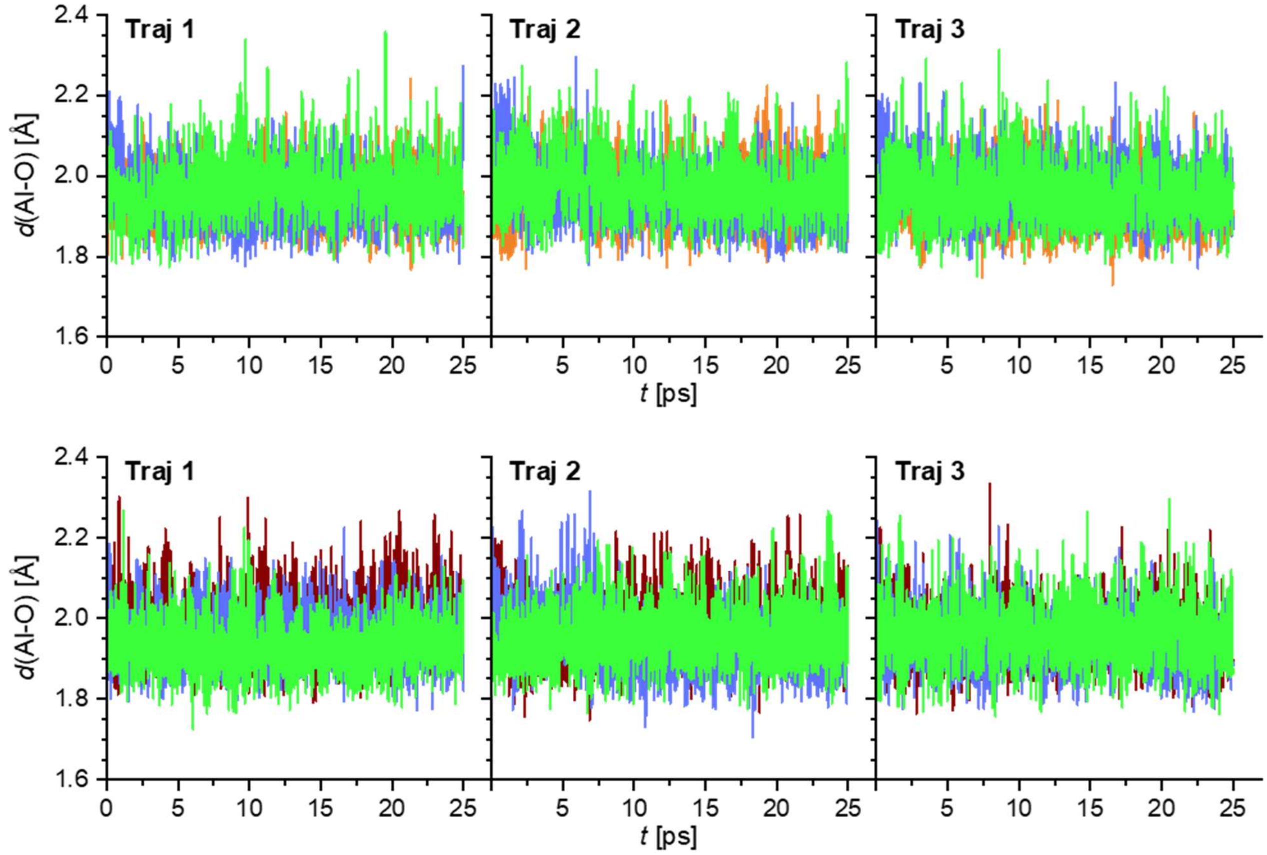

Figure S4: Time evolution of Al−O distances during AIMD simulations of 5-FU@FAU_fwAl$_{oct}$_2Al models with 14 additional non-coordinated H_2O molecules. Top: 1_O4 configuration, bottom: 2_O2 configuration. The color scheme follows **Figure 6**.

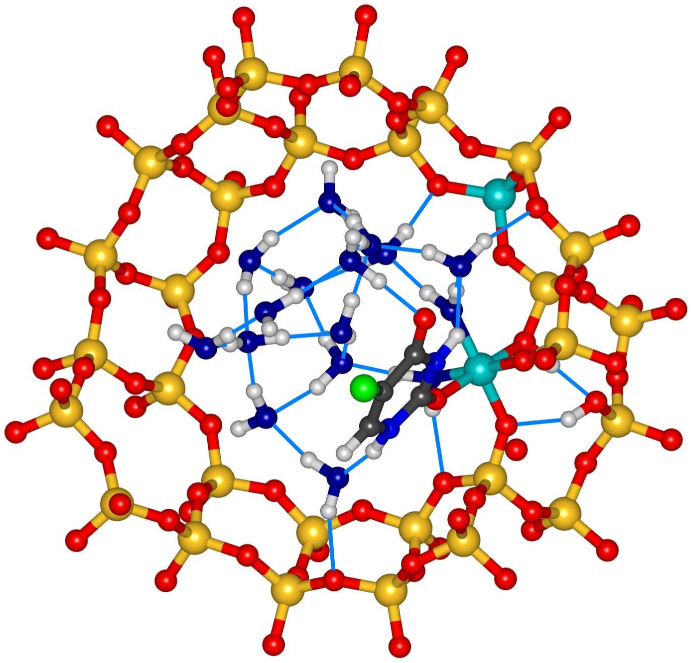

Figure S5: Representative DFT-optimized snapshot taken from AIMD simulation of 5-FU@FAU_fwAl$_{oct}$_2Al with 14 additional non-coordinated H_2O molecules.

S2.5 DFT-calculated "composite" IR spectra

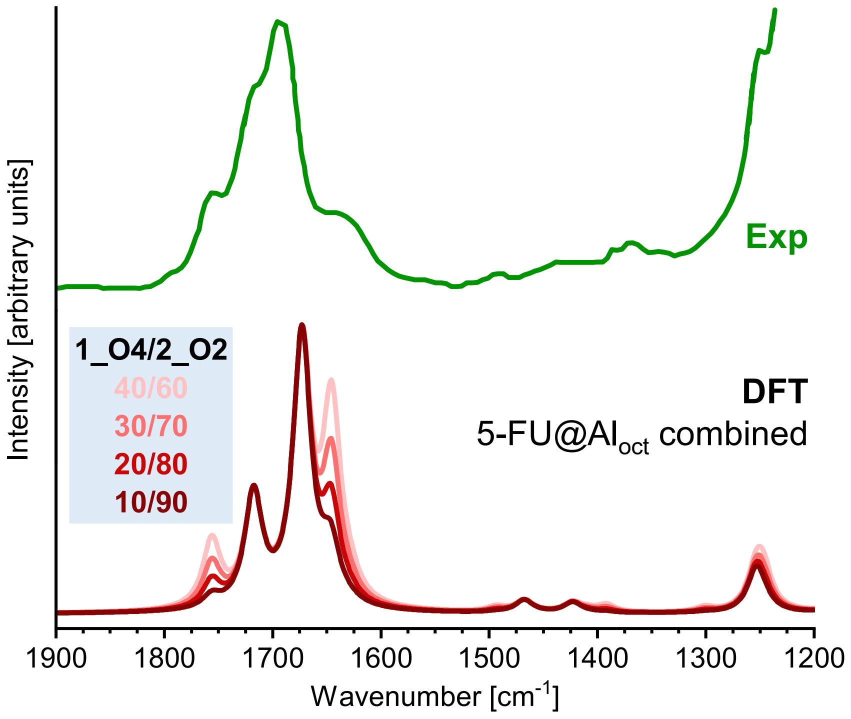

Figure S6: Pink and red curves: IR spectra obtained by combining the two DFT-calculated IR spectra shown in **Figure 5**, assuming different percentages of the relative contribution from the 1_O4 configuration (40% to 10%) and from the 2_O2 configuration (60% to 90%, see inset). The experimental spectrum is shown for comparison (green).

S2.6 Results of DFT-GIPAW calculations

Table S5: ^{27}Al NMR isotropic chemical shifts obtained from DFT-GIPAW calculations.

**Model**	**$\delta_{iso}(Al_{tet})$ [ppm]**	**$\delta_{iso}(Al_{oct})$ [ppm]**
H-FAU_1H	57.1	Not applicable
5-FU@H-FAU_1H	55.8	Not applicable
FAU_fwAl$_{oct}$_2Al*	56.9	1.6
FAU_fwAl$_{oct}$_2Al + 6 H_2O*	57.8	2.0
5-FU@FAU_fwAl$_{oct}$_2Al (5-FU_1_O4)	58.6	−6.6
5-FU@FAU_fwAl$_{oct}$_2Al (5-FU_2_O2)	56.3	−2.7
5-FU@FAU_fwAl$_{oct}$_2Al (5-FU_1_O4) + 14 H_2O*	59.0	−7.6
5-FU@FAU_fwAl$_{oct}$_2Al (5-FU_2_O2) + 14 H_2O*	56.1	−8.5
FAU_fwAl$_{tbp}$_2Al	56.5	24.7**
FAU_fwAl$_{oct}$_2Al after AIMD, dissociated Al−O(H_2O) bond*	55.6	27.9**
5-FU@FAU_fwAl$_{oct}$_2Al (1_O4) after AIMD, dissociated Al−O(H_2O) bond*	56.2	20.8**

* Lowest-energy structure obtained from DFT optimization of AIMD snapshot

** Trigonal-bipyramidal coordination of Al

References

[1] H. Sun, S. J. Mumby, J. R. Maple, A. T. Hagler, *J. Am. Chem. Soc.* **1994**, *116*, 2978–2987.

[2] M. Waldman, A. T. Hagler, *J. Comput. Chem.* **1993**, *14*, 1077–1084.

[3] F. S. Emami, V. Puddu, R. J. Berry, V. Varshney, S. V. Patwardhan, C. C. Perry, H. Heinz, *Chem. Mater.* **2014**, *26*, 2647–2658.

[4] M. Fischer, *CrystEngComm* **2024**, *26*, 3795–3807.

[5] C. J. Pickard, F. Mauri, *Phys. Rev. B* **2001**, *63*, 245101.

[6] J. R. Yates, C. J. Pickard, F. Mauri, *Phys. Rev. B* **2007**, *76*, 024401.

[7] S. J. Clark, M. D. Segall, C. J. Pickard, P. J. Hasnip, M. I. J. Probert, K. Refson, M. C. Payne, *Zeitschrift für Krist.* **2005**, *220*, 567–570.

[8] V. Milman, K. Refson, S. J. Clark, C. J. Pickard, J. R. Yates, S.-P. Gao, P. J. Hasnip, M. I. J. Probert, A. Perlov, M. D. Segall, *J. Mol. Struct. THEOCHEM* **2010**, *954*, 22–35.

[9] J. P. Perdew, K. Burke, M. Ernzerhof, *Phys. Rev. Lett.* **1996**, *77*, 3865–3868.

[10] D. Willimetz, A. Erlebach, C. J. Heard, L. Grajciar, *Digit. Discov.* **2025**, *4*, 275–288.

[11] A. Datt, E. A. Burns, N. A. Dhuna, S. C. Larsen, *Microporous Mesoporous Mater.* **2013**, *167*, 182–187.

[12] J. D. Gale, A. L. Rohl, *Mol. Simul.* **2003**, *29*, 291–341.

[13] M. J. Sanders, M. Leslie, C. R. A. Catlow, *J. Chem. Soc. Chem. Commun.* **1984**, 1271–1273.
